# Phosphide Delivery to a Cyclotrisilene**

**DOI:** 10.1002/anie.201409908

**Published:** 2014-11-21

**Authors:** Thomas P Robinson, Michael J Cowley, David Scheschkewitz, Jose M Goicoechea

**Affiliations:** Department of Chemistry, University of Oxford, Chemistry Research Laboratory12 Mansfield Road, Oxford, OX1 3TA (UK); Chair in General and Inorganic Chemistry, Saarland University66125 Saarbrücken (Germany)

**Keywords:** 2-phosphaethynolate, main-group chemistry, multiple bonds, silenes, silicon

## Abstract

The reactivity of the 2-phosphaethynolate anion (PCO^−^) towards a cyclic trisilene (cSi_3_(Tip)_4_) is reported. The result is the net activation of the P=C and Si=Si multiple bonds of the precursors affording a heteroatomic bicyclo[1.1.1]pentan-2-one analogue ([P(CO)Si_3_(Tip)_4_]^−^; **1**). This reaction can be interpreted as the formal addition of a phosphide and a carbonyl across the Si=Si double bond. Photolytic decarbonylation of **1** results in the incorporation of the phosphide vertex into the cyclotrisilene scaffold, yielding a congener of the cyclobutene anion with considerable allylic character.

Unsaturated silicon species are transient intermediates during the gas-phase deposition of elemental silicon from molecular precursors.[1] Cyclotrisilene, *c*Si_3_H_4_, is the simplest unsaturated cyclic silane, and to date it has only been observed in the gas phase. Kinetic and thermodynamic stabilization of cyclotrisilenes using bulky silyl substituents was initially achieved by the groups of Kira[2] and Sekiguchi[3] (Scheme 1). More recently, Scheschkewitz and co-workers reported the synthesis of the first aryl substituted cyclotrisilenes and their reactivity towards N-heterocyclic carbenes.[4]

**Scheme 1:**
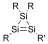
Reported cyclotrisilenes. R=*t*Bu_2_MeSi, R′=Si(*t*Bu_2_MeSi)_3_;[2] R=R′=*t*Bu_2_MeSi;[3] R=R′=Tip (2,4,6-triisopropylphenyl); R=Tip, R′=Cp*.[4]

In view of the importance of phosphorus as an n-dopant for silicon-based semiconductors,[5] its incorporation into stable silicon compounds is both of theoretical and practical interest. While saturated species containing silicon and phosphorus in the same molecule are abundant,[6] unsaturated species featuring both elements are relatively rare. Notable examples include phosphasilenes (P=Si)[7] and phosphino disilenes.[7g, 8] In particular the transfer of phosphorus to unsaturated silicon rings and clusters without concomitant incorporation of additional organic substituents is an attractive prospect for the introduction of an n-dopant at the molecular level.

Cyclotrisilenes have been shown to undergo ring expansion with isocyanides[9] and carbon monoxide.[10] Consequently, the use of unsaturated phosphorus-containing small molecules appeared to be a viable strategy for the desired incorporation of phosphorus into an unsaturated silicon scaffold. Grützmacher and co-workers have recently employed 2-phosphaethynolate (PCO^−^; a phosphorus-containing analogue of cyanate) as a phosphide source for the synthesis of a base-coordinated phosphinidene.[11] We therefore decided to explore the reactivity of *c*Si_3_(Tip)_4_ towards the 2-phosphaethynolate anion. The latter species was first reported by Becker and co-workers,[12] and has received significant attention following the development of new methods for its formation.[13]–[15] A handful of studies have already shown that PCO^−^ reacts with unsaturated substrates including heteroallenes, such as carbodiimides, isocyanates, and CO_2_.[14]–[17]

Reaction of [K(18-crown-6)][PCO] with *c*Si_3_(Tip)_4_ gives rise to two products as determined by ^31^P NMR spectroscopy (each exhibiting a singlet resonance).[18] The relative ratio of these two species, and the chemical shift of the downfield resonance, are strongly dependent on solvent polarity and are also influenced by addition of different cation sequestering agents. When the reaction is carried out in a polar solvent, for example, THF, two resonances are observed in the ^31^P NMR spectrum at about −56 and −323 ppm in a 1:2 ratio. Conversely, in the presence of non-polar solvents (benzene and toluene), the product with the downfield ^31^P NMR resonance is favored (typically in a 4:1 ratio). It is worth noting that no interconversion between the products is observed over time or on heating the reaction mixtures. Addition of 2,2,2-cryptand to the two reagents followed by dissolution in THF further favors the formation of the product at −323.0 ppm (**1**), presumably by disrupting solution phase ion-pair contacts. This species was isolated as a compositionally pure sample in moderate yields (35 %) and crystallized as bright orange crystals from hot benzene. In the ^29^Si NMR spectrum, it exhibits three doublets at −1.8, −10.9, and −16.1 ppm. The chemical shifts of the resonances, and the magnitudes of the silicon–phosphorus coupling constants, indicate that the resonance at −1.8 ppm corresponds to a saturated Si(Tip)_2_ moiety at two bonds from the phosphorus atom; the more upfield resonances are consistent with Si(Tip) groups with single bond coupling to a phosphorus nucleus. In the IR spectrum, a band arising from a bridging carbonyl bond stretch was observed at 1584 cm^−1^. The negative ion mode electrospray mass spectrum reveals a molecular-ion mass-envelope at 955.80 Da in line with a 1:1 adduct of PCO^−^ and cyclotrisilene (an additional, lower intensity, mass-envelope arising from the loss of a carbonyl was also observed at *m*/*z* 927.75). These data firmly support the formation of [P(CO)Si_3_(Tip)_4_]^−^ (**1**, Scheme 2).

**Scheme 2:**
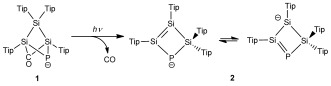
Photolysis of 1 to afford 2.

The single-crystal X-ray analysis of the product, [K(2,2,2-crypt)]**1**⋅2 C_6_H_6_ reveals complete cleavage of the P=C multiple bond of the 2-phosphaethnynolate anion (Figure 1). The PCO^−^ anion has added across the Si=Si double bond as a phosphide (P^−^) and a carbonyl moiety (CO) resulting in a bicyclo[1.1.1]pentanone-type structure. Saturated bicyclo[1.1.1]pentane units have been extensively employed as repeat units of [*n*]staffanes;[19] persila[*n*]staffanes have been reported recently by Iwamoto and co-workers.[20] Of the heavier bicyclo[1.1.1]pentanones, a derivative with two silicon atoms in the scaffold has also been described which is free of any additional functionality aside of the carbonyl moiety.[21] In the case of **1**, the presence of the anionic phosphide type vertex, which is consistent with the upfield ^31^P NMR chemical shift, constitutes a potentially valuable site for further functionalization. The interatomic distance between Si1 and Si2 (formerly the double bond of the cyclotrisilene) of 2.618(1) Å (c.f. 2.118(1) Å in *c*Si_3_(Tip)_4_)[4a] is indicative of the expected absence of a strong direct interaction between the bridgehead silicon atoms. The two remaining Si=Si bonds (both 2.375(1) Å) each display typical single bond lengths.[22] Greater variation is observed for the P=Si bonds, which adopt values of 2.174(4) and 2.215(4) Å. This slight asymmetry may arise from the crystallographic disorder of the phosphide and carbonyl moieties over the two bridging positions (each at 50 % occupancy). The optimized geometry of **1** calculated using a dispersion-corrected functional (ωB97XD/6-311g(d,p); with polarized continuum solvent model for THF) was found to be in good agreement with experimental values as were the computed NMR chemical shifts (see the Supporting Information for full details).[23] The mechanism of formation for **1** remains obscure at this time; fast reaction rates prevented us from identifying any intermediates. Previous studies on the reactivity of *c*Si_3_(Tip)_4_ with unsaturated compounds such as isocyanides, however, suggest that a [2+1] cycloaddition product may be involved.[9] Conversely, PCO^−^ has been shown to give rise to [2+2] cycloaddition products with isocyanates and ketenes, so a different reaction pathway involving the formation of a five-membered ring cannot be excluded either.[15]

**Figure 1:**
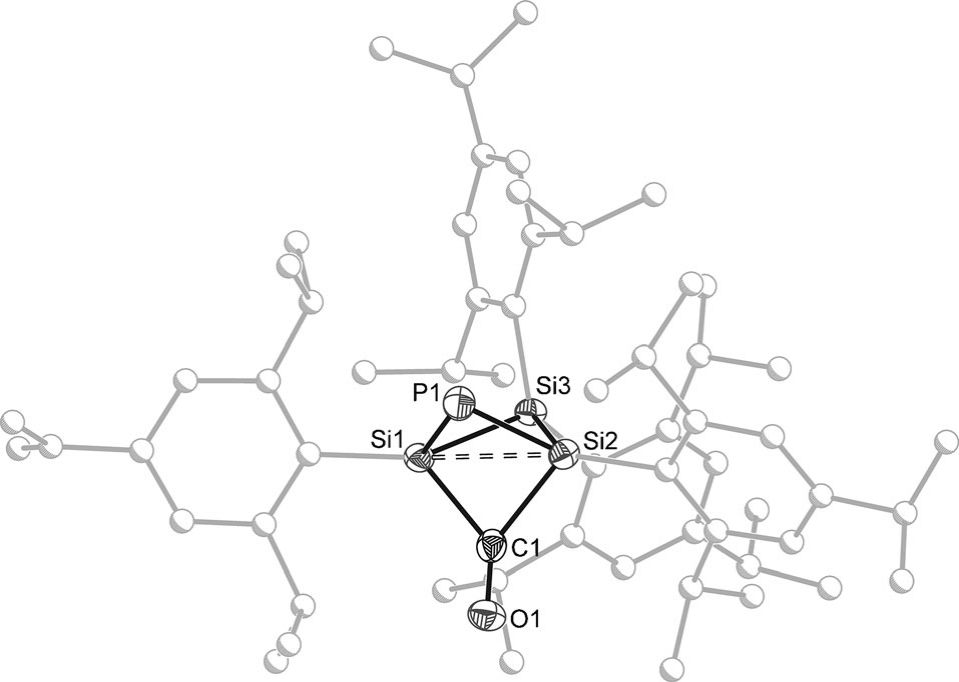
Molecular structure of the anionic moiety in [K(2,2,2-crypt))]1⋅2 C_6_H_6_ (ellipsoids set at 50 % probability; hydrogen atoms, crystal solvent, and second component of positional disorder of phosphide vertex and carbonyl (1:1) omitted for clarity). Atoms of Tip groups shown as spheres of arbitrary radius. Selected interatomic distances [Å] and angles [°]: P1–Si1 2.215(4), P1–Si2 2.174(4), Si1⋅⋅⋅Si2 2.618(1), Si1–Si3 2.375(1), Si2–Si3 2.375(1), Si1–C1 2.009(15), Si2–C1 2.005(16), C1–O1 1.204(15); Si1-P1-Si2 73.22(11), Si1-C1-Si2 81.4(6), P1-Si1-Si3 94.16(9), P1-Si1-C1 82.7(4), P1-Si2-Si3 95.20(9), P1-Si2-C1 83.9(4), Si1-Si3-Si2 66.89(2).

UV irradiation of a solution of [K(2,2,2-crypt)]**1** is accompanied by a notable color change (from orange to blue–green) and quantitatively affords a new compound with a singlet resonance in the ^31^P NMR spectrum. For preparative purposes, the same product (albeit with a different countercation) can be isolated by photolyzing a mixture of [K(18-crown-6)][PCO] and *c*Si_3_(Tip)_4_ in toluene, affording a compound with a ^31^P NMR resonance at −93.9 ppm. This corresponds to the lowest field resonance observed from the crude reaction between PCO^−^ and *c*Si_3_(Tip)_4_ in non-polar solvents. Notably, the characteristic ^13^C NMR signal of the carbonyl moiety in **1** at 222.7 ppm disappears, suggesting decarbonylation of **1** on irradiation. This is further corroborated by electrospray mass-spectrometric measurements, which reveal the molecular ion as [PSi_3_(Tip)_4_]^−^ (observed at 928.11 Da). The ^1^H NMR spectrum confirmed the presence of one molecule of 18-crown-6 in the product, which was thus tentatively identified as [K(18-crown-6)]**2** (Scheme 2). The net reaction between PCO^−^ and *c*Si_3_(Tip)_4_ under photolytic conditions may be considered as a direct incorporation of a phosphide into an unsaturated ring system. As already mentioned (see above), the use of the 2-phosphaethynolate anion as a phosphide anion transfer agent has previously been reported by Grützmacher and co-workers to yield an N-heterocyclic carbene-stabilized phosphinidene.[11]

It is worth noting that the ^31^P NMR chemical shift of [K(18-crown-6)]**2** is strongly dependent on the nature of the solvent: while it appears at −56.6 ppm in [D_8_]THF, it is observed at −93.9 ppm in [D_8_]toluene. This effect may arise from solvent coordination to **2** or the separation of ion pairs in solution. There is a large upfield shift observed for the ^29^Si NMR resonance arising from the β-silicon (with respect to the phosphide center) of the formal silicon–silicon double bond, which was observed at −39.9 ppm (^2^*J*_Si–P_=35.4 Hz) (consistent with significant delocalization of negative charge onto this atom, as represented in the second resonance form in Scheme 2). While the resonance of the saturated silicon atom was recorded at −14.9 ppm (^1^*J*_Si-P_=72.5 Hz), the resonance for the remaining unsaturated silicon center is found far downfield at 193.1 ppm (^1^*J*_Si–P_=138.2 Hz). Strongly polarized Si=Si bonds with their characteristically wide dispersion of ^29^Si NMR shifts have been subject to detailed experimental and computational studies.[24], [25]

The UV/Vis absorption spectrum of [K(18-crown-6)]**2** in THF reveals its longest wavelength absorption at significantly lower energy than in case of **1** (**1**: *λ*_max_=490 nm; **2**: *λ*_max_=594 nm) consistent with the blue–green color of the product. TDDFT calculations on the optimized structure of the contact ion pair [K(18-crown-6)]**2**_calc_ (ωB97XD/6-311g(d,p); with polarized continuum solvent model for THF)[23] quantitatively reproduce the trend although the signals are considerably blue-shifted (**1**_calc_: *λ*_max_=437; [K(18-crown-6)]**2**_calc_: *λ*_max_=514 nm). As expected the longest wavelength transition in [K(18-crown-6)]**2**_calc_ is largely dominated by the HOMO–LUMO transition (94 %), which corresponds to the π and π* orbitals of the Si=Si=P moiety. The calculated chemical shifts of optimized **2**_calc_ reasonably agree with experimental values, and are independent of the existence (or absence) of a contact ion pair (see the Supporting Information).

Dark-green crystals of **2** were grown from toluene (as [K(18-crown-6)]**2**⋅0.5 C_7_H_8_) and characterized by single-crystal X-ray diffraction (Figure 2). The anionic moiety consists of a slightly folded PSi_3_ four-membered ring (folding angle between P–Si1–Si2/Si1–Si2–Si3 24.056(24)°). The structure is consistent with a significant allylic character across the unsaturated atoms in the ring (resonance forms in Scheme 2), which is manifested in the interatomic bond distances. Consequently, P1=Si1 is notably (0.11 Å) shorter than P1=Si2 (2.156(1) and 2.263(1) Å, respectively), as expected for a phospha-allylic system. The Si1=Si3 distance of 2.212(1) Å is longer than the typical Si=Si double bond, but very similar to that of a related cyclotetrasilenide anion reported by Sekiguchi and co-workers.[26] The delocalization of the negative charge over P1, Si1, and Si3 is also apparent in the upfield ^29^Si chemical shift and the pyramidalization of the Si3 center (Σ_angles_=332.64°). It is located 0.610(1) Å above the plane defined by Si1, Si2, and the *ipso*-carbon atom of the Tip substituent to which it is bonded.

**Figure 2:**
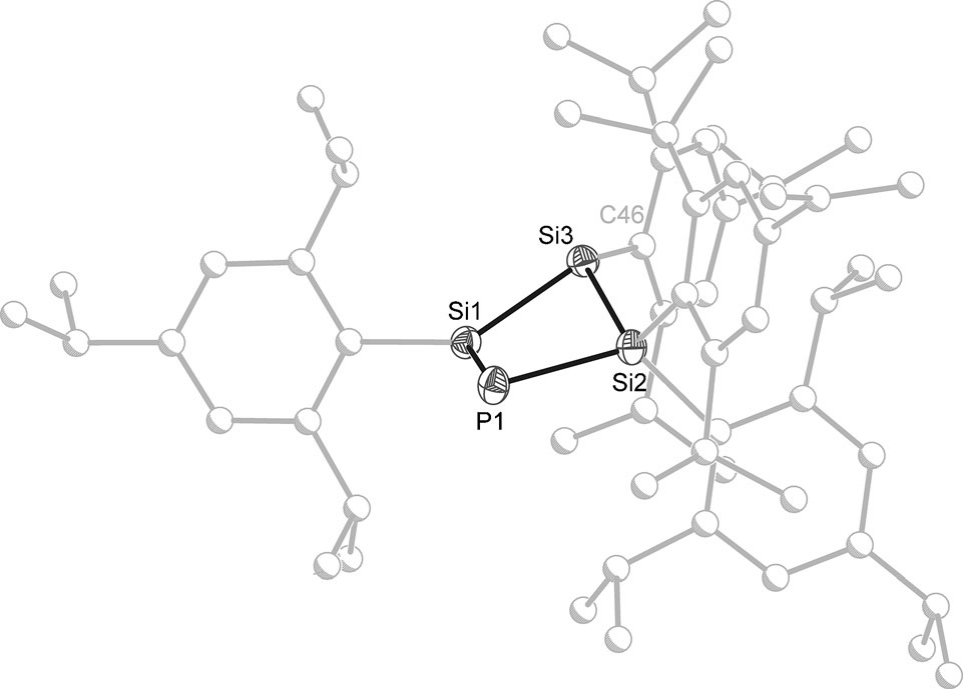
Molecular structure of the anionic moiety in [K(18-crown-6)]2⋅0.5 C_7_H_8_ (ellipsoids set at 50 % probability; hydrogen atoms are omitted for clarity; atoms of Tip groups are pictured as spheres of arbitrary radius. Selected interatomic distances [Å] and angles [°]: P1–Si1 2.156(1), P1–Si2 2.263(1), Si1–Si3 2.212(1), Si2–Si3 2.364(1); Si1-P1-Si2 80.785(16), P1-Si1-Si3 101.669(18), P1-Si2-Si3 94.108(16), Si1-Si3-Si2 77.427(15), Si1-Si3-C46 128.46(4), Si1-Si3-C46 126.75(4).

In conclusion, we report the incorporation of a phosphide into an unsaturated silicon scaffold. The reaction of the highly versatile cyclotrisilene *c*Si_3_(Tip)_4_ with potassium phosphaethynolate under photolytic conditions and loss of carbon monoxide results in a formal ring expansion to yield an anionic heavier cyclobutene analogue. The intermediate prior to decarbonylation was isolated and already displays a completely cleaved phosphorus–carbon bond, the extreme case of P=C bond activation. The reactivity of both compounds in particular regarding the nucleophilic phosphide functionality and the formal Si=Si moiety is currently being investigated in our laboratories.

Dedicated to Professor Gerhard Roewer on occasion of his 75th birthday
