# Supplementary material for: Phosphide Delivery to a Cyclotrisilene
Source: Angew Chem Int Ed Engl. 2014 Nov 21;54(2):683–6. doi: 10.1002/anie.201409908 (PMC4506556; doi:10.1002/anie.201409908)
Supplement: Supplementary file 1 [file anie0054-0683-sd1.pdf]

Angewandte Chemie
Eine Zeitschrift der Gesellschaft Deutscher Chemiker

Supporting Information

© Wiley-VCH 2014

69451 Weinheim, Germany

Phosphide Delivery to a Cyclotrisilene**

Thomas P. Robinson, Michael J. Cowley, David Scheschkewitz, and Jose M. Goicoechea**

anie_201409908_sm_miscellaneous_information.pdf

Supporting Information

Contents

1. Experimental section

2. Single crystal X-ray diffraction data

3. NMR spectra

4. ESI-MS spectra

5. UV-Vis spectra

6. IR spectrum

7. Computation details and MO-plots

8. References

1. Experimental section

General synthetic methods. All reactions and product manipulations were carried out under an inert atmosphere of argon or dinitrogen using standard Schlenk-line or glovebox techniques (MBraun UNIlab glovebox maintained at < 0.1 ppm H_2O and < 0.1 ppm O_2). [K(18-crown-6)][PCO] and *c*$Si_3(Tip)_4$ were synthesized according to a previously reported synthetic procedure.[1,2] 2,2,2-crypt (4,7,13,16,21,24-hexaoxa-1,10-diazabicyclo[8,8,8]-hexacosane; VWR, 99%) was used as received after careful drying under vacuum. Hexane (hex; Sigma Aldrich HPLC grade), pentane (pent; Sigma Aldrich HPLC grade) benzene (Rathburn, HPLC grade), toluene (Sigma Aldrich HPLC grade) were purified using an MBraun SPS-800 solvent system. Tetrahydrofuran (THF; ≥99.9%, Sigma Aldrich) was distilled over a sodium metal/benzophenone mixture. [D_8]-THF (Euriostop, 99.5%) and [D_8]-tol (Sigma Aldrich, 99.6%) were dried over CaH_2 and vacuum distilled before use. All dry solvents were stored under argon in gas-tight ampoules. Additionally hexane, pentane, benzene, toluene and THF were stored over activated 3 Å molecular sieves.

Synthesis of [K(2,2,2-crypt)][Si_3Tip_4PCO] ([K(2,2,2-crypt)][**1**]). [K(18-crown-6)][PCO] (150 mg, 0.414 mmol) and 2,2,2-crypt (156 mg, 0.414 mmol) were combined in a Schlenk tube and dissolved in THF (5 mL). A solution of *c*$Si_3(Tip)_4$ (372 mg, 0.414 mmol) in THF (10 mL) was subsequently added. The resulting dark orange mixture was stirred for two hours after which all volatiles were removed *in vacuo*. The residue was washed with cold hexanes (3 × 5 mL). The remaining solid was extracted into toluene and filtered *via* cannula. The toluene was removed *in vacuo* and the oily residue extracted into hot hexane and filtered immediately. Upon standing at room temperature an orange precipitate of [K(2,2,2-crypt)][**1**] formed, which was isolated by decanting the supernatant solution. The product was purified by crystallisation from hot benzene. Yield: 208 mg (37%). Calculated for $C_{79}H_{128}KN_2O_7PSi_3$: C 69.17, H 9.40, N 2.04. Observed: C 66.99, H 9.02, N 2.08. Samples were found to be

recurrently and reproducibly deficient in carbon. $^{31}P\{^{1}H\}$ (202.38 MHz, [D$_8$]-THF, 298 K): δ (ppm) −323.0 ($^{1}J_{P-Si}$ satellites = 97.9 Hz). $^{29}Si\{^{1}H\}$ (99.3 MHz, [D$_8$]-THF, 298 K,): δ (ppm) −1.8 (d, $^{2}J_{Si-P}$ = 19.6 Hz), −10.9 (d, $^{1}J_{Si-P}$ = 97.9 Hz), −16.1 (d, $^{1}J_{Si-P}$ = 94.9 Hz). ^{1}H NMR (500 MHz, [D$_8$]THF, 298 K): δ (ppm) 7.05 (d, $^{4}J_{H-H}$ = 1.73 Hz, 1H; Ar-CH), 6.90 (br s, 2H; Ar-CH), 6.84 (v br s, 2H; Ar-CH), 6.71 (d, $^{4}J_{H-H}$ = 1.46 Hz, 1H; Ar-CH), 6.63 (d, $^{4}J_{H-H}$ = 1.73 Hz, 1H; Ar-CH), 6.57 (d, $^{4}J_{H-H}$ = 1.63 Hz, 1H; Ar-CH), 4.99 (v br s, 1H; *i*Pr-CH), 4.63 (sept, $^{3}J_{H-H}$ = 6.4 Hz, 1H; *i*Pr-CH), 4.49 (sept, $^{3}J_{H-H}$ = 6.4 Hz, 1H; *i*Pr-CH), 4.05 (sept, $^{3}J_{H-H}$ = 6.4 Hz, 1H; *i*Pr-CH), 3.56 (sept, $^{3}J_{H-H}$ = 6.7 Hz, 1H; *i*Pr-CH), 3.45 (s, 12H; 2,2,2-crypt), 3.42−3.40 (m, 12H; 2,2,2-crypt), 3.31 (sept, $^{3}J_{H-H}$ = 6.7 Hz, 1H; *i*Pr-CH), 3.12 (sept, $^{3}J_{H-H}$ = 6.7 Hz, 1H; *i*Pr-CH), 2.80−2.63 (m, 4H; 5 × *i*Pr-CH), 2.43−2.41 (m, 12H; 2,2,2-crypt), 1.75 (d, $^{3}J_{H-H}$ = 6.7 Hz, 3H; *i*Pr-CH$_3$), 1.54 (d, $^{3}J_{H-H}$ = 6.4 Hz, 3H; *i*Pr-CH$_3$), 1.51 (d, $^{3}J_{H-H}$ = 6.7 Hz, 3H; *i*Pr-CH$_3$), 1.31 (v br s, 6H; 2 × *i*Pr-CH$_3$), 1.28 (d, $^{3}J_{H-H}$ = 6.7 Hz, 3H; *i*Pr-CH$_3$), 1.25 (d, $^{3}J_{H-H}$ = 6.4 Hz, 3H; *i*Pr-CH$_3$), 1.18−1.10 (m, 30H; 10 × *i*Pr-CH$_3$), 1.04 (d, $^{3}J_{H-H}$ = 6.4 Hz, 3H; *i*Pr-CH$_3$), 0.56 (d, $^{3}J_{H-H}$ = 6.7 Hz, 3H; *i*Pr-CH$_3$), 0.47 (d, $^{3}J_{H-H}$ = 6.4 Hz, 3H; *i*Pr-CH$_3$), 0.34 (d, $^{3}J_{H-H}$ = 6.4 Hz, 3H; *i*Pr-CH$_3$), 0.23 (v br s, 3H; *i*Pr-CH$_3$), 0.09 (d, $^{3}J_{H-H}$ = 6.4 Hz, 3H; *i*Pr-CH$_3$), −0.01 (d, $^{3}J_{H-H}$ = 6.4 Hz, 3H; *i*Pr-CH$_3$). ^{1}H NMR (499.9 MHz, [D$_8$]-THF, 253 K): δ (ppm) 7.04 (s, 1H; Ar-CH), 6.92 (s, 1H; Ar-CH), 6.90 (s, 2H; Ar-CH), 6.74 (s, 1H; Ar-CH), 6.71 (s, 1H; Ar-CH), 6.64 (s, 1H; Ar-CH), 6.57 (s, 1H; Ar-CH), 4.98 (br sept, $^{3}J_{H-H}$ = 6.4 Hz, 1H; *i*Pr-CH), 4.60 (sept, $^{3}J_{H-H}$ = 6.4 Hz, 1H; *i*Pr-CH), 4.48 (sept, $^{3}J_{H-H}$ = 6.7 Hz; 1H, *i*Pr-CH), 4.01 (sept, $^{3}J_{H-H}$ = 6.4 Hz, 1H; *i*Pr-CH), 3.50 (sept, $^{3}J_{H-H}$ = 6.4 Hz, 1H; *i*Pr-CH), 3.44 (s, 12H; 2,2,2-crypt), 3.40−3.39 (m, 12H; 2,2,2-crypt), 3.29 (sept, $^{3}J_{H-H}$ = 6.7 Hz, 1H; *i*Pr-CH), 3.09 (sept, $^{3}J_{H-H}$ = 6.4 Hz, 1H; *i*Pr-CH), 2.78−2.62 (m, 4H; 5 × *i*Pr-CH), 2.40 (br s, 12H; 2,2,2-crypt), 1.74 (d, 3H; *i*Pr-CH$_3$, obscured by solvent), 1.55 (d, $^{3}J_{H-H}$ = 6.4 Hz, 3H; *i*Pr-CH$_3$), 1.49 (d, $^{3}J_{H-H}$ = 6.4 Hz, 3H; *i*Pr-CH$_3$), 1.41 (d, $^{3}J_{H-H}$ = 6.1 Hz, 3H; *i*Pr-CH$_3$), 1.33 (d, $^{3}J_{H-H}$ = 6.1 Hz, 3H; *i*Pr-CH$_3$), 1.26 (d, $^{3}J_{H-H}$ = 6.7 Hz, 3H; *i*Pr-CH$_3$), 1.24−1.20 (m,

6H; 2 × *i*Pr-CH_3), 1.20−1.08 (m, 27H; 10 × *i*Pr-CH_3), 1.02 (d, $^3J_{H–H}$ = 6.4 Hz, 3H; *i*Pr-CH_3), 0.54 (d, $^3J_{H–H}$ = 6.7 Hz, 3H; *i*Pr-CH_3), 0.45 (d, $^3J_{H–H}$ = 6.7 Hz, 3H; *i*Pr-CH_3), 0.32 (d, $^3J_{H–H}$ = 6.7 Hz, 3H; *i*Pr-CH_3), 0.20 (d, $^3J_{H–H}$ = 6.4 Hz, 3H; *i*Pr-CH_3), 0.05 (d, $^3J_{H–H}$ = 6.7 Hz, 3H; *i*Pr-CH_3), −0.06 (d, $^3J_{H–H}$ = 6.4 Hz, 3H; *i*Pr-CH_3). $^{13}C\{^1H\}$ (125.8 MHz, [D_8]-THF, 298 K): δ (ppm) 222.7 (d, $^2J_{C–P}$ = 15.7 Hz; C=O), 156.7 (br; Tip-C), 156.3 (Tip-C), 156.2 (Tip-C), 155.7 (Tip-C), 154.7 (Tip-C), 153.2 (Tip-C), 152.3 (Tip-C), 148.1 (Tip-C), 147.6 (Tip-C), 147.4 (Tip-C), 146.7 (Tip-C), 142.2 (Tip-C), 138.5 (Tip-C), 137.6 (d, $^{ts}J_{C–P}$ = 6.5 Hz; Tip-C), 135.8 (Tip-C), 122.0 (Tip-CH), 121.9 (Tip-CH), 121.5 (Tip-CH), 120.4 (Tip-CH), 120.2 (Tip-CH), 118.9 (Tip-CH), 71.0 (2,2,2-crypt), 68.2 (2,2,2-crypt), 54.5 (2,2,2-crypt), 39.3 (*i*Pr-CH), 36.3 (*i*Pr-CH), 35.9 (br, *i*Pr-CH), 35.8 (*i*Pr-CH), 35.2 (*i*Pr-CH), 35.1 (*i*Pr-CH), 35.1 (*i*Pr-CH), 35.0 (*i*Pr-CH), 35.0 (*i*Pr-CH), 34.2 (*i*Pr-CH), 30.8 (d, $^{ts}J_{C–P}$ = 11.4 Hz; *i*Pr-CH_3), 29.6 (*i*Pr-CH_3), 29.4 (d, $^{ts}J_{C–P}$ = 11.4 Hz; ^{i}Pr-CH_3), 28.8 (v br; ^{i}Pr-CH_3), 27.7 (*i*Pr-CH_3), 25.4* (*i*Pr-CH_3), 24.7 (*i*Pr-CH_3), 24.5 (*i*Pr-CH_3), 24.4 (*i*Pr-CH_3), 24.3 (*i*Pr-CH_3), 24.2 (*i*Pr-CH_3), 24.1 (*i*Pr-CH_3), 23.4 (*i*Pr-CH_3), 21.3 (*i*Pr-CH_3). (* Obscured by solvent). IR (Nujol): υ(CO) = 1584 cm^{-1}. UV-Vis (THF): $\lambda_{max}(\varepsilon)$ = 490 nm (1462), 348 nm (10147), 282 nm (32930). ESI-MS (THF, negative mode): *m*/*z* 927.75 [Si_3Tip_4P]$^-$ (18%), 955.80 [Si_3Tip_4PCO]$^-$ (100%*). No mass envelopes observed in the positive mode.

Note: The ^1H NMR spectrum reveals a dynamic process for the two Tip groups of the $Si(Tip)_2$ centre, not unlike the one previously reported for *c*$Si_3(Tip)_4$. This fluxional behaviour was resolved at 253 K (however extensive overlap of resonances in the VT ^1H NMR spectra precludes the calculation of activation parameters).

*Synthesis of [K(18-crown-6)][Si_3Tip_4P] ([K(18-crown-6)][**2**]).* [K(18-crown-6)][PCO] (75 mg, 0.207 mmol) and *c*$Si_3(Tip)_4$ (185 mg, 0.207 mmol) were combined in an air-tight ampoule and toluene (15 mL) was added at –30 °C. The solution was maintained at –30 °C

for 2 hours after which it was slowly warmed to room temperature and stirred for a further 12 hours. The resulting blue/green solution contains a mixture of compounds **1** and **2** in a ratio of *ca.* 1:4. To completely drive the reaction towards the formation of **2**, the solution was irradiated using a 600 W broadband UV lamp for *ca*. 6 hours with progress monitored by ^{31}P NMR spectroscopy. When conversion was complete the solution was filtered *via* cannula and all volatiles were removed *in vacuo* affording a green/blue oil. The oil was dissolved in pentane (10 mL) with minimal stirring and left to stand at room temperature for 12 hours yielding dark blue crystals of [K(18-crown-6)][Si_3Tip_4P], which were isolated by decanting the supernatant solution. Yield = 115 mg (45%). Diffraction quality single-crystals were grown from toluene at –35 °C. Calculated for $C_{72}H_{116}KO_6PSi_3$: C 70.19, H 9.49, N 0.00. Observed: C 70.06, H 9.35, N 0.00. ^{31}P{^{1}H} (202.4 Hz, [D$_8$]-THF, 298 K,): δ (ppm) −56.6 ($^1J_{P–Si}$ satellites = 138.2 Hz, $^1J_{P–Si}$ satellites = 72.5 Hz). ^{29}Si{^{1}H} (99.3 MHz, [D$_8$]-THF, 298 K): δ 193.1 (d, $^1J_{Si–P}$ = 138.2 Hz), −14.9 (d, $^1J_{Si–P}$ = 72.5 Hz), −39.9 (d, $^2J_{Si–P}$ = 35.4 Hz). ^{31}P{^{1}H} (202.4 Hz, [D$_8$]-Tol, 298 K): δ (ppm) −93.9 ($^1J_{P–Si}$ = 129.2 Hz, $^1J_{P–Si}$ = 65.6 Hz). ^{29}Si{^{1}H} (99.3 MHz, [D$_8$]-Tol, 298 K): δ (ppm) 187.7 (d, $^1J_{Si–P}$ = 128.8 Hz), −13.9 (d, $^1J_{Si–P}$ = 65.1 Hz), −22.6 (d, $^2J_{Si–P}$ = 38.2 Hz). ^{1}H NMR (499.9 MHz, [D$_8$]-THF, 298 K): δ (ppm) 7.00 (br, 1H; Ar-CH) , 6.90 (br, 1H; Ar-CH), 6.83 (s, 2H; Ar-CH), 6.65 (br s, 4H; Ar-CH), 6.48 (br, 1H; Ar-CH), 5.91 (br, 1H; *i*Pr-CH), 4.79 (v br, 2H; *i*Pr-CH), 4.71 (sept, $^3J_{H–H}$ = 6.7 Hz, 2H; *i*Pr-CH), 4.18 (br, 1H; *i*Pr-CH), 3.53 (v br, 2H; *i*Pr-CH), 3.51 (s, 24H; 18-crown-6), 2.74 (sept, $^3J_{H–H}$ = 6.9 Hz, 1H; *i*Pr-CH), 2.67 (br sept, 2H; *i*Pr-CH), 2.62 (sept, $^3J_{H–H}$ = 6.9 Hz, 1H; *i*Pr-CH), 1.50–1.21 (br, 3H; *i*Pr-CH_3), 1.43 (br, 6H; *i*Pr-CH_3), 1.36 (br, 6H; *i*Pr-CH_3), 1.28 (br, 12H; *i*Pr-CH_3), 1.21–0.99 (br, 9H; *i*Pr-CH_3), 1.18 (d, $^3J_{H–H}$ = 6.9 Hz, 6H; *i*Pr-CH_3), 1.14 (br, d, 12H; *i*Pr-CH_3), 1.08 (d, $^3J_{H–H}$ = 6.9 Hz, 6H; *i*Pr-CH_3), 0.76 (v br, 3H; *i*Pr-CH_3), 0.30 (br, 3H; *i*Pr-CH_3), 0.20 (br, 3H; *i*Pr-CH_3), 0.03 (br, 3H, *i*Pr-CH_3). ^{1}H NMR (499.9 MHz, [D$_8$]-THF, 233 K): δ (ppm) 7.00 (s, 1H; Tip-CH), 6.89 (s, 1H; Tip-CH), 6.83 (s, 2H; Tip-

CH), 6.74 (broad s, 1H; Tip-CH), 6.63 (s, 2H; Tip-CH), 6.54 (broad s, 1H; Tip-CH), 6.46 (s, 1H; Tip-CH), 5.88 (sept, $^3J_{H–H}$ = 6.5 Hz, 1H; *i*Pr-CH), 5.46 (broad s, 1H; *i*Pr-CH), 4.69 (broad m, 2H; *i*Pr-CH), 4.17 (sept, $^3J_{H–H}$ = 6.5 Hz, 1H; *i*Pr-CH), 4.08 (broad s, 1H; *i*Pr-CH), 3.56–3.43 (m, 2H, *i*Pr-CH), 3.51 (s, 24H, 18-crown-6), 2.74 (sept, $^3J_{H–H}$ = 6.9 Hz, 1H; *i*Pr-CH), 2.71–2.62 (m, 2H; *i*Pr-CH), 2.62 (sept, $^3J_{H–H}$ = 6.9 Hz, 1H; *i*Pr-CH), 1.42 (d, $^3J_{H–H}$ = 6.5 Hz, 6H; *i*Pr-CH_3), 1.36–1.33 (m, 6H; *i*Pr-CH_3), 1.31 (d, $^3J_{H–H}$ = 6.5 Hz, 6H; *i*Pr-CH_3), 1.26 (d, $^3J_{H–H}$ = 6.9 Hz, 6H; *i*Pr-CH_3), 1.17 (d, $^3J_{H–H}$ = 6.9 Hz, 6H; *i*Pr-CH_3), 1.16–1.04 (m, 27H; *i*Pr-CH_3), 0.85 (br s, 3H; *i*Pr-CH_3), 0.27 (d, $^3J_{H–H}$ = 6.5 Hz, 3H; *i*Pr-CH_3), 0.16 (d, $^3J_{H–H}$ = 6.5 Hz, 3H; *i*Pr-CH_3), 0.14 (broad s, 3H; *i*Pr-CH_3), –0.02 (d, $^3J_{H–H}$ = 6.45 Hz, 3H; *i*Pr-CH_3). $^{13}C\{^1H\}$ (125.8 MHz, [D_8]-THF, 238 K): δ (ppm) 156.0 (br, Tip-*C*), 155.8 (Tip-*C*), 154.1 (Tip-*C*), 153.9 (Tip-*C*), 153.4 (Tip-*C*), 153.2(Tip-*C*), 148.1 (Tip-*C*), 147.0 (Tip-*C*), 146.9 (Tip-*C*), 145.7 (d, $J_{C–P}$ = 7.0 Hz, Tip-*C*), 145.4 (Tip-*C*), 145.2 (Tip-*C*), 144.5 (Tip-*C*), 141.9 (Tip-*C*), 122.3 (Tip-*C*H), 120.6 (Tip-*C*H), 120.5 (br, Tip-*C*H), 120.2 (Tip-*C*H), 119.9 (Tip-*C*H), 119.8 (Tip-*C*H), 70.9 (crown-*C*H), 37.4 (br, *i*Pr-*C*H), 35.7 (*i*Pr-*C*H), 35.5 (*i*Pr-*C*H), 35.2 (*i*Pr-*C*H), 35.1 (br, *i*Pr-*C*H), 35.1 (*i*Pr-*C*H), 34.1 (*i*Pr-*C*H), 33.9 (*i*Pr-*C*H), 31.4 (d, $J_{C–P}$ = 10.8 Hz), 28.0 (*i*Pr-CH_3), 27.0 (*i*Pr-CH_3), 26.5 (*i*Pr-CH_3), 26.3 (*i*Pr-CH_3), 26.2 (*i*Pr-CH_3), 26.1 (*i*Pr-CH_3), 25.4* (*i*Pr-CH_3), 24.9 (*i*Pr-CH_3), 24.9 (*i*Pr-CH_3), 24.7 (*i*Pr-CH_3), 24.6–24.5 (m, *i*Pr-CH_3), 24.5 (*i*Pr-CH_3), 24.2 (*i*Pr-CH_3), 23.9 (*i*Pr-CH_3), 23.5 (*i*Pr-CH_3), 22.3 (*i*Pr-CH_3) (* Obscured by solvent). UV-Vis (THF): $\lambda_{max}(\varepsilon)$ = 594 nm (4697), 362 nm (7556). ESI-MS (THF, negative mode): *m*/*z* 928.11 $[Si_3Tip_4P]^-$ (100%*).

Single crystal X-ray structure determination: Single-crystal X-ray diffraction data were collected using either an Oxford Diffraction Supernova dual-source diffractometer equipped with a 135 mm Atlas CCD area detector. Crystals were selected under Paratone-N oil, mounted on micromount loops and quench-cooled using an Oxford Cryosystems open flow

N_2 cooling device. Data were collected at 150 K using mirror monochromated Cu K_α radiation (λ = 1.5418 Å; Oxford Diffraction Supernova) and processed using the CrysAlisPro package, including unit cell parameter refinement and inter-frame scaling (which was carried out using SCALE3 ABSPACK within CrysAlisPro).[3] Equivalent reflections were merged and diffraction patterns processed with the CrysAlisPro suite. Structures were subsequently solved using direct methods and refined on F^2 using the SHELXL 2014-3 package.[4]

Additional characterization techniques: ^{1}H, ^{13}C, ^{31}P and ^{29}Si NMR spectra were acquired at 499.9, 125.8, 202.4 and 99.2 MHz, respectively, on a Bruker AVIII 500 MHz NMR Spectrometer. ^{1}H and ^{13}C NMR spectra were referenced to the most downfield solvent resonance (^{1}H NMR [D$_8$]-THF: δ = 3.58 ppm; ^{13}C NMR [D$_8$]THF: δ = 67.2 ppm). ^{31}P and ^{29}Si spectra were externally referenced to an 85% solution of H_3PO_4 in H_2O (δ = 0 ppm) and TMS (δ = 0 ppm), respectively.

Positive and negative ion mode electrospray ionization mass spectra were recorded on DMF solutions (10–20 µM) on a Waters LCT Time of Flight mass spectrometer with a Z-spray source (150°C source temperature, 200°C desolvation temperature, 2.4 kV capillary voltage and 25 V cone voltage). The samples were made up inside a glovebox under an inert atmosphere and rapidly transferred to the spectrometer in an air-tight syringe. Samples were introduced directly with a 1 mL SGE syringe and a syringe pump at 0.6 mL h^{-1}.

Elemental analyses were carried out by Elemental Microanalyses Ltd. (Devon, U.K.). Samples (approx. 5 mg) were submitted in sealed Pyrex ampoules.

2. Single crystal X-ray diffraction data

Table S1. Selected X-ray data collection and refinement parameters for [K(2,2,2-crypt)][**1**]·$2C_6H_6$ and [K(18-crown-6))][**2**]·$0.5C_7H_8$.

	[K(2,2,2-crypt)][**1**]·$2C_6H_6$	[K(18-crown-6)][**2**]·$0.5C_7H_8$
Formula	$C_{91}H_{140}KN_2O_7PSi_3$	$C_{75.5}H_{120}KO_6PSi_3$
Fw [g mol^{-1}]	1528.38	1278.05
crystal system	monoclinic	monoclinic
space group	$P2_1/c$	$I\,2/a$
a (Å)	19.9221(9)	24.4411(10)
b (Å)	17.6488(7)	27.2214(4)
c (Å)	26.6643(8)	25.0883(17)
β (°)	102.440(3)	110.113(4)
V (Å^3)	9155.1(6)	15673.8(13)
Z	4	8
radiation, λ (Å)	Cu K_α (1.54178)	
T (K)	150(2)	
ρ_{calc} (g cm^{-3})	1.109	1.083
μ (mm^{-1})	1.439	1.576
reflections collected	93580	128663
independent reflections	16124	16356
parameters	1041	834
R(int)	0.0458	0.0372
R1/wR2,[a] I ≥ 2σI (%)	5.89/15.06	3.73/9.79
R1/wR2,[a] all data (%)	7.01/15.78	4.28/10.34
GOF	1.051	1.030

[a] $R1 = [\Sigma||F_o| - |F_c||]/\Sigma|F_o|$; $wR2 = \{[\Sigma w[(F_o)^2 - (F_c)^2]^2]/[\Sigma w(F_o^2)^2]\}^{1/2}$; $w = [\sigma^2(F_o)^2 + (AP)^2 + BP]^{-1}$, where $P = [(F_o)^2 + 2(F_c)^2]/3$ and the A and B values are 0.0740 and 6.97 for [K(2,2,2-crypt)][**1**]·$2C_6H_6$ and 0.0512 and 11.68 for [K(18-crown-6)][**2**]·$0.5C_7H_8$.

3. NMR spectra

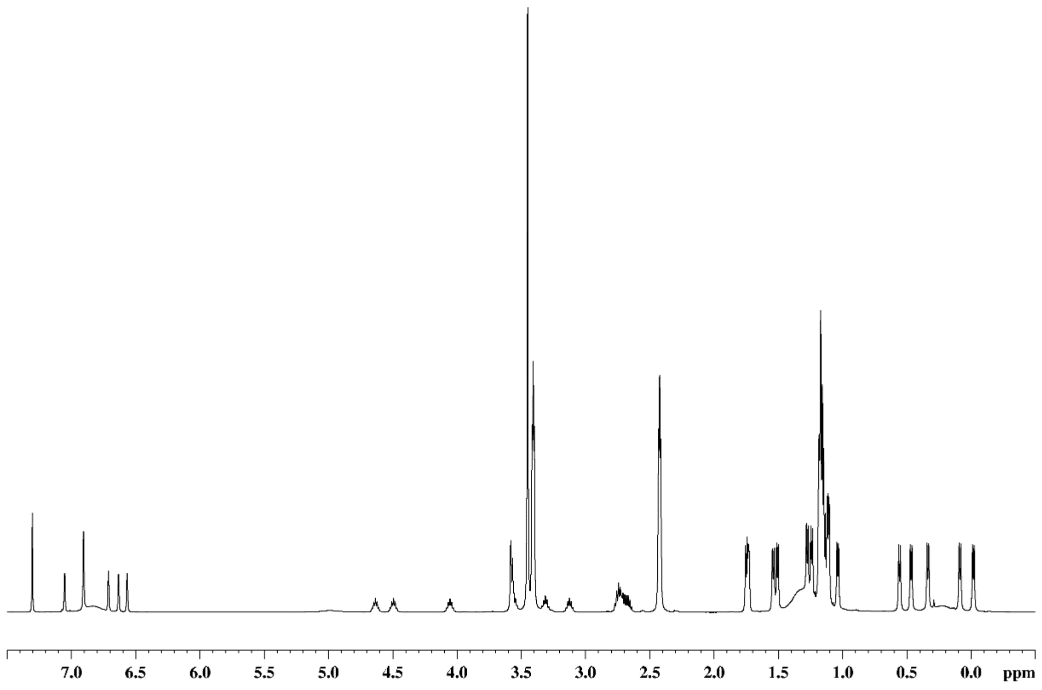

Figure S1. Room temperature ^{1}H NMR spectrum of [K(2,2,2-crypt)][**1**].

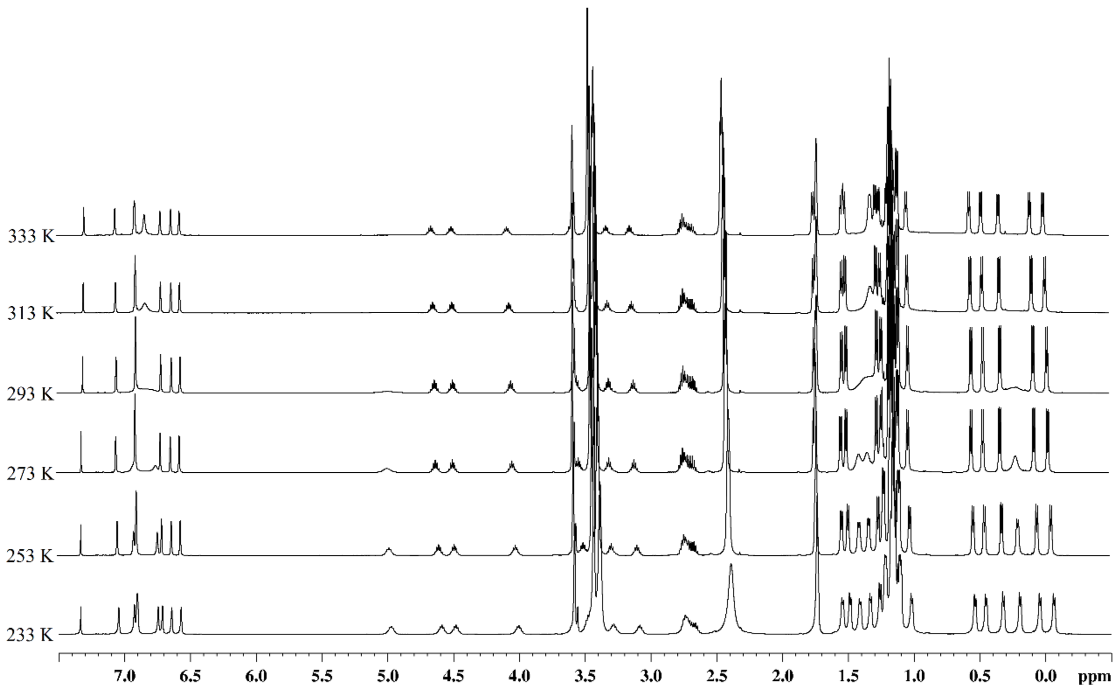

Figure S2. Variable temperature ^{1}H NMR spectra of [K(2,2,2-crypt)][**1**] ([D_8]-THF).

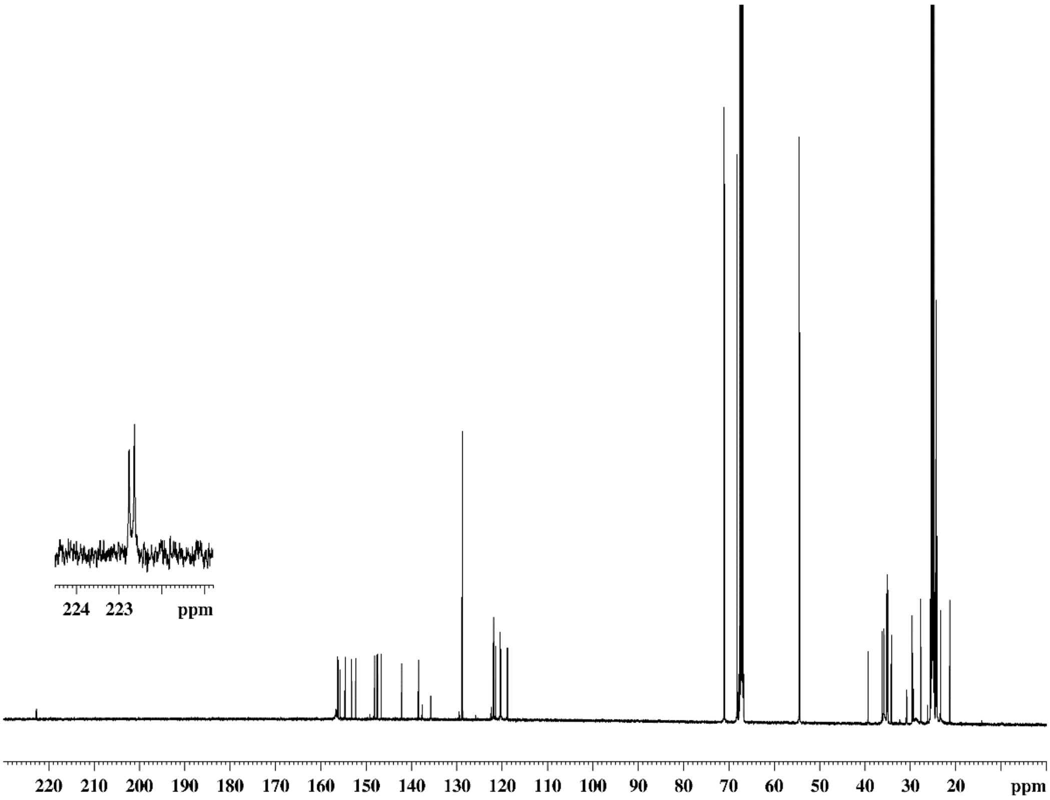

Figure S3. Room temperature $^{13}C\{^{1}H\}$ NMR spectrum of [K(2,2,2-crypt)][**1**].

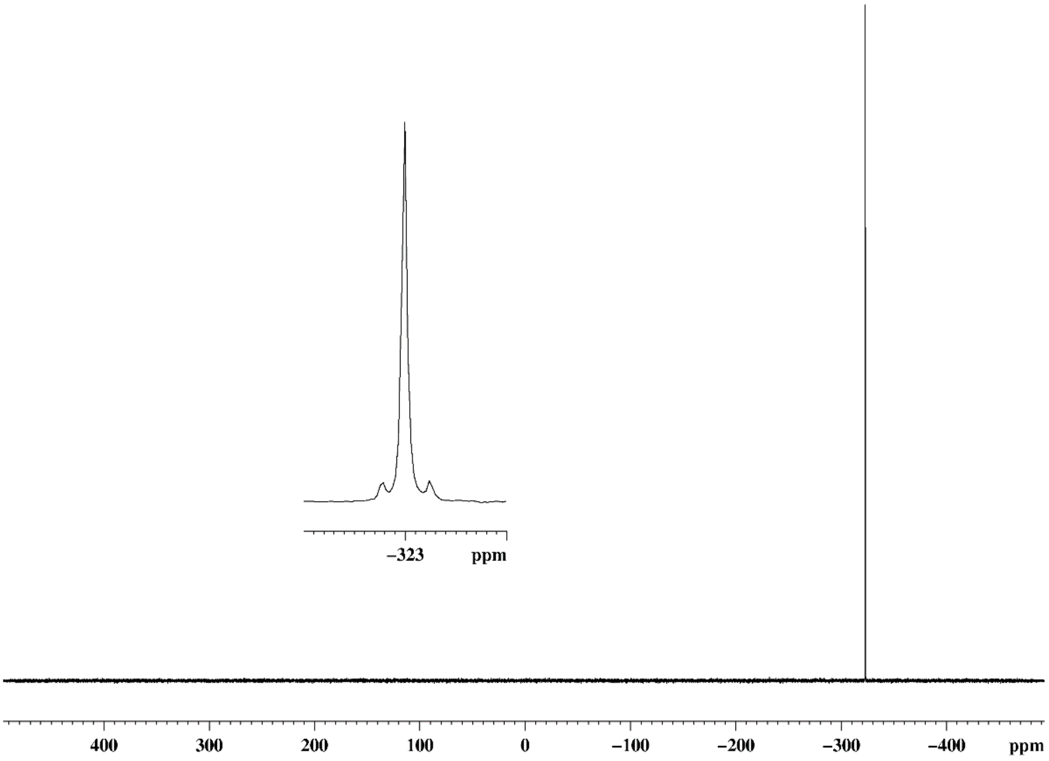

Figure S4. Room temperature $^{31}P\{^{1}H\}$ NMR spectrum of [K(2,2,2-crypt)][**1**].

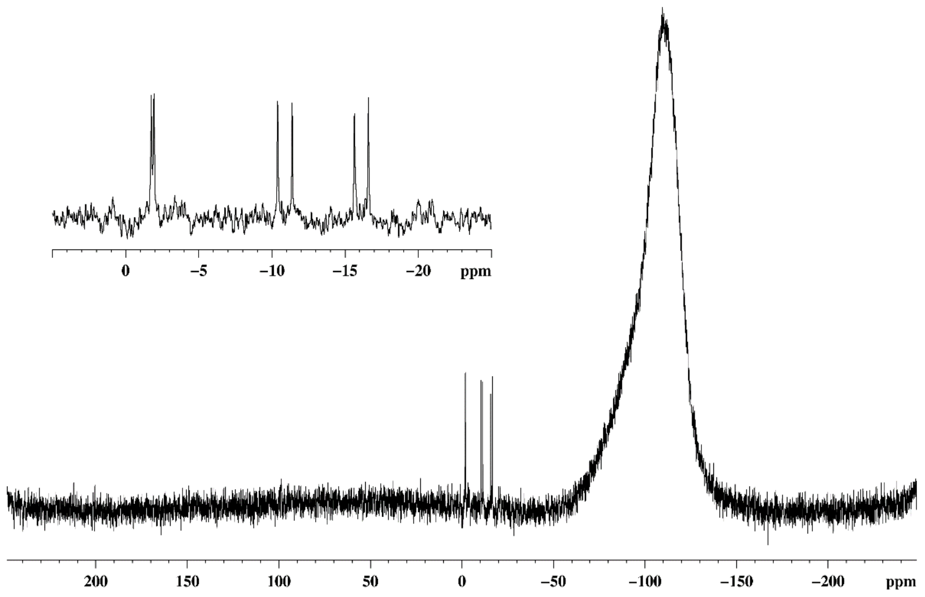

Figure S5. Room temperature $^{29}Si\{^1H\}$ NMR spectrum of [K(2,2,2-crypt)][**1**].

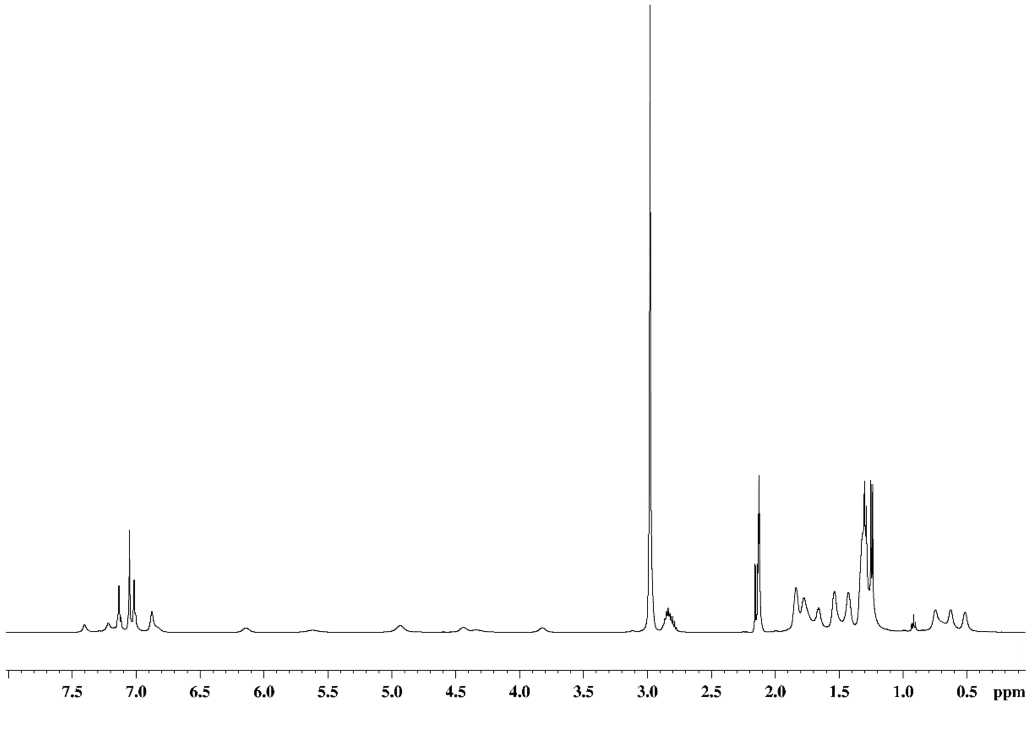

Figure S6. Room temperature ^1H NMR spectrum of [K(18-crown-6)][**2**] ([D_8]-tol).

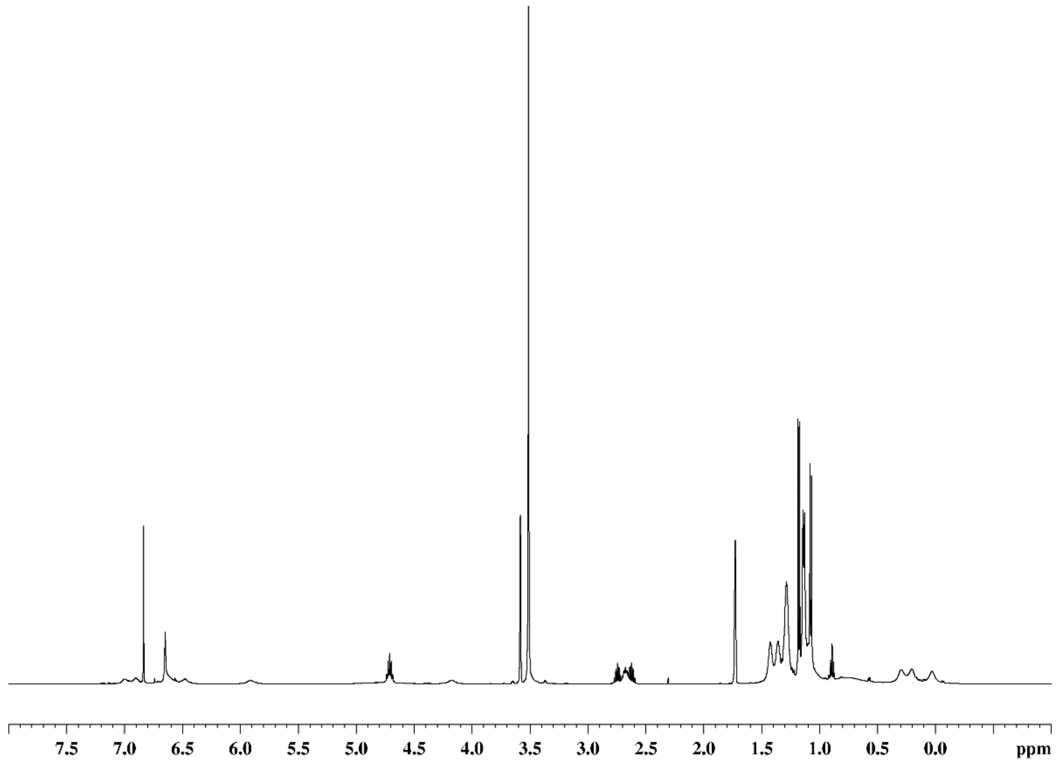

Figure S7. Room temperature ^{1}H NMR spectrum of [K(18-crown-6)][**2**] at ([D$_8$]-THF).

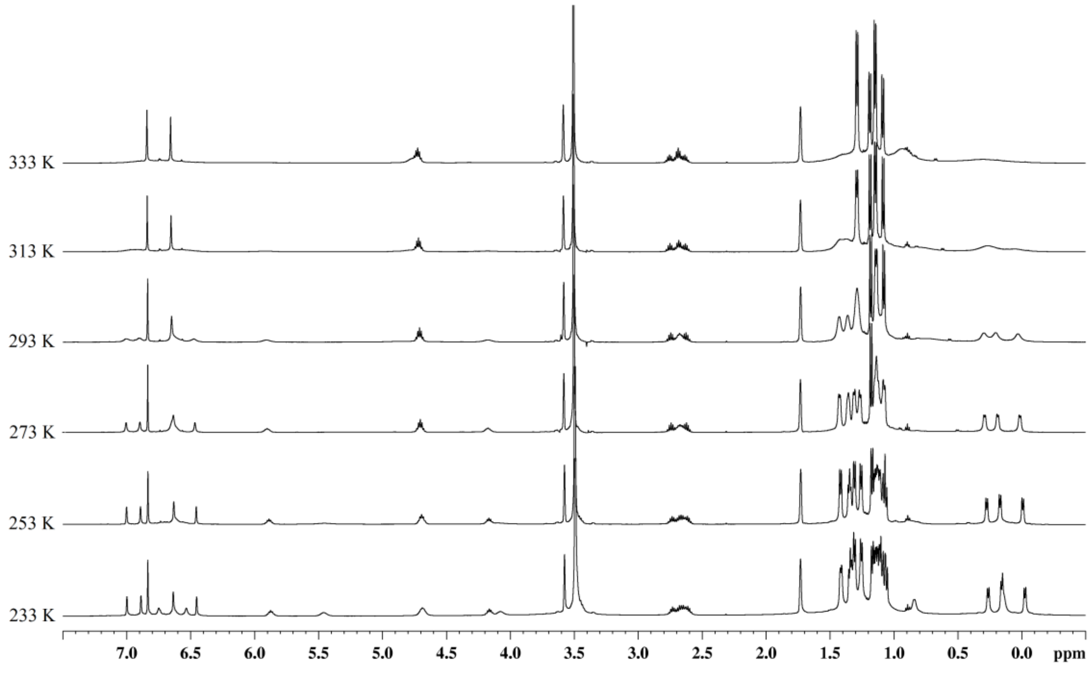

Figure S8. VT ^{1}H NMR spectra of [K(18-Crown-6)][**2**] ([D$_8$]-THF).

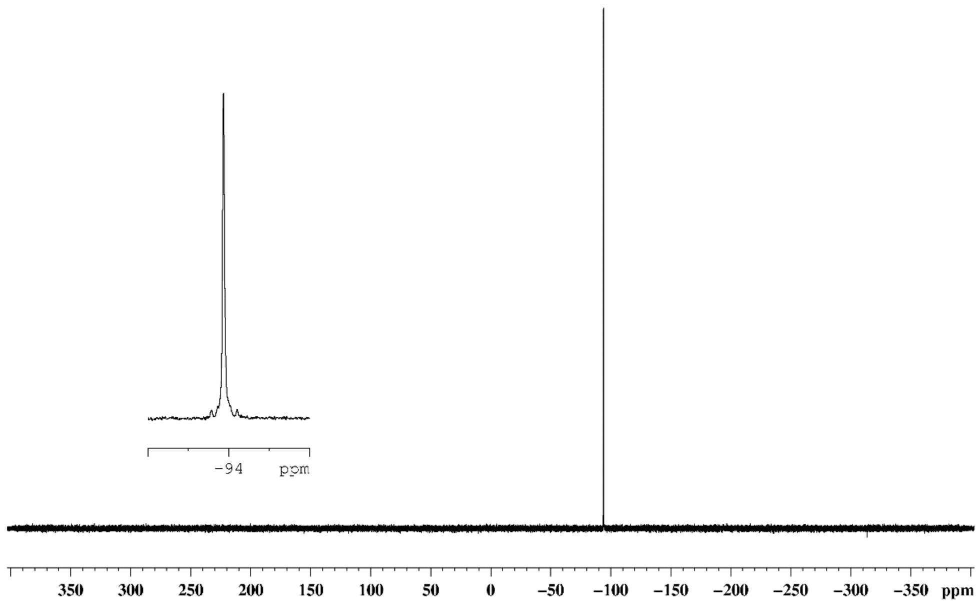

Figure S9. Room temperature $^{31}P\{^{1}H\}$ NMR spectrum of [K(18-crown-6)][**2**] ([D_8]-tol).

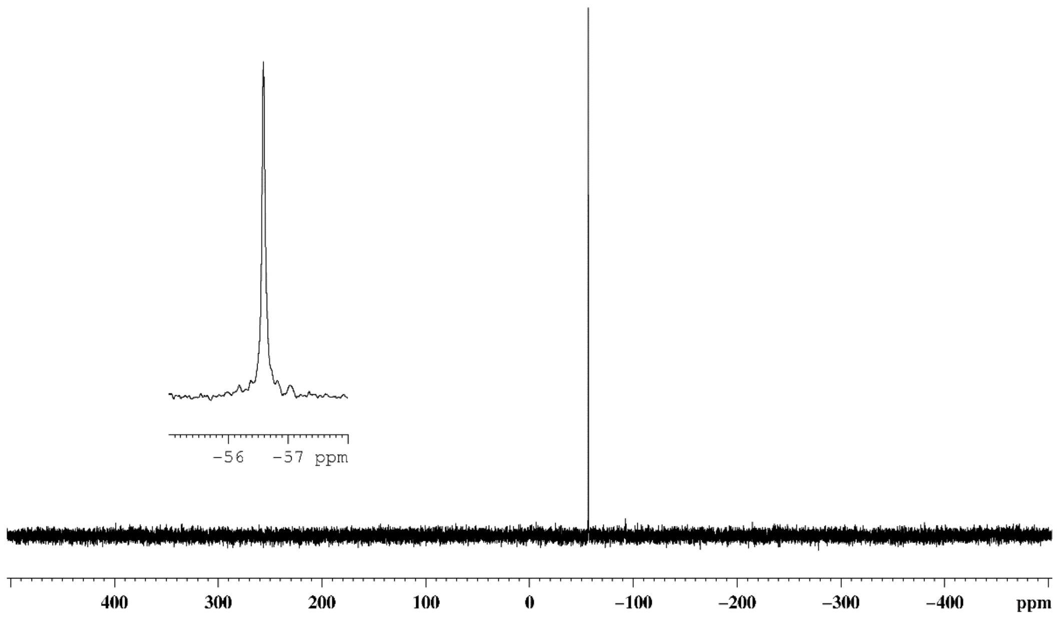

Figure S10. Room temperature $^{31}P\{^{1}H\}$ NMR spectrum of [K(18-crown-6)][**2**] ([D_8]-THF).

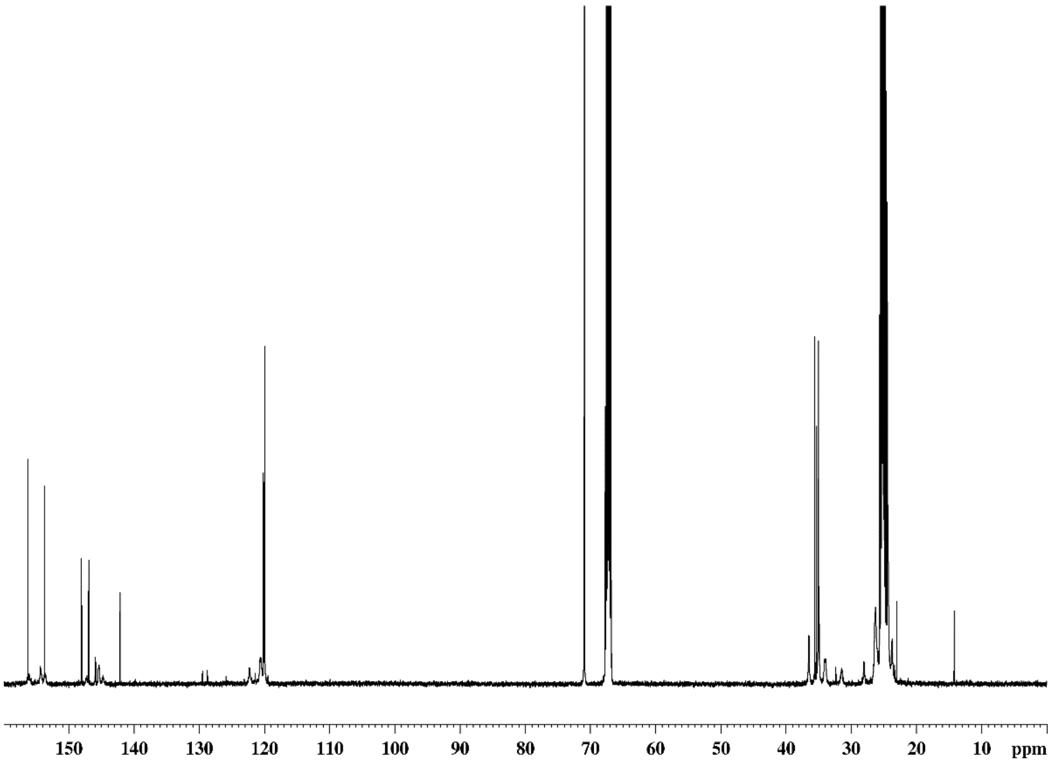

Figure S11. Room temperature $^{13}C\{^{1}H\}$ NMR spectrum of [K(18-crown-6)][**2**] ([D_8]-THF).

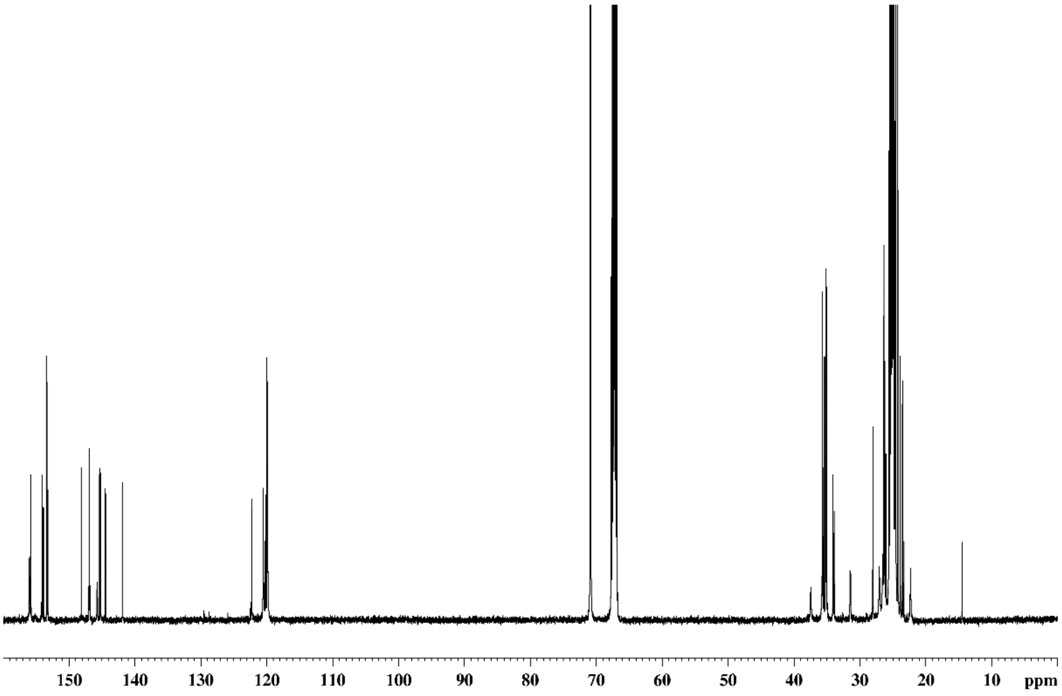

Figure S12. $^{13}C\{^{1}H\}$ NMR spectrum of [K(18-crown-6)][**2**] at 238 K ([D_8]-THF).

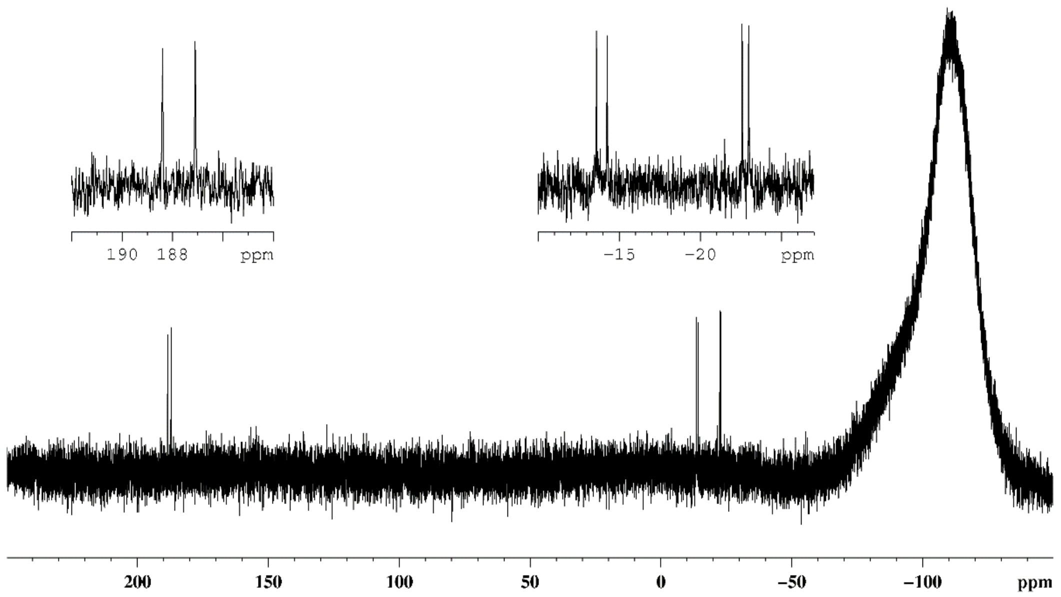

Figure S13. $^{29}Si\{^1H\}$ NMR spectrum of [K(18-crown-6)][**2**] at 238 K ([D8]-THF).

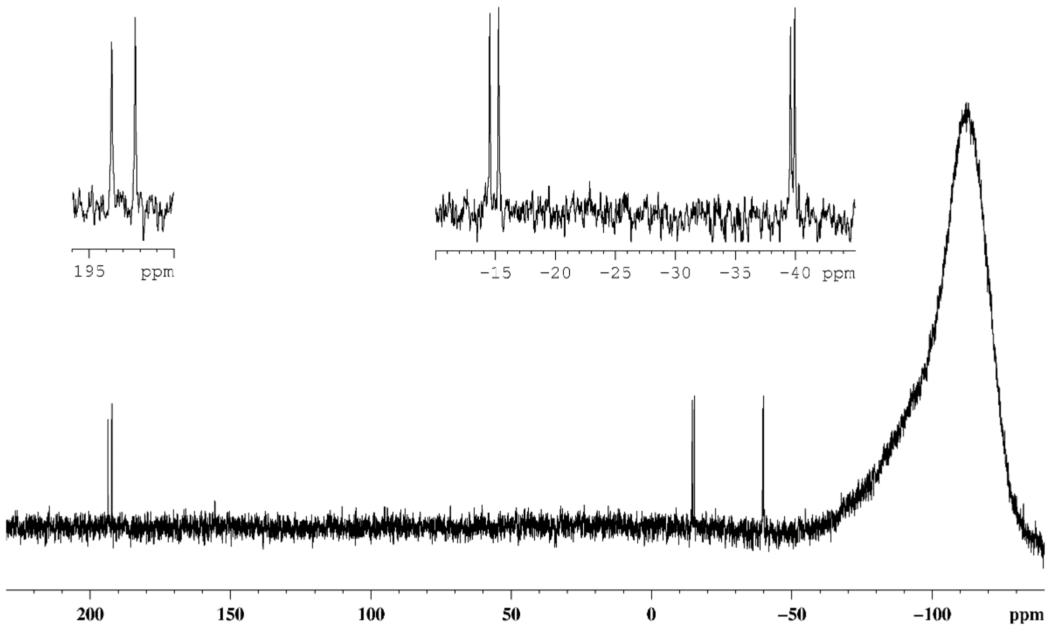

Figure S14. $^{29}Si\{^1H\}$ NMR spectrum of [K(18-crown-6)][**2**] at 238 K ([D8]-THF).

4. ESI-MS spectra

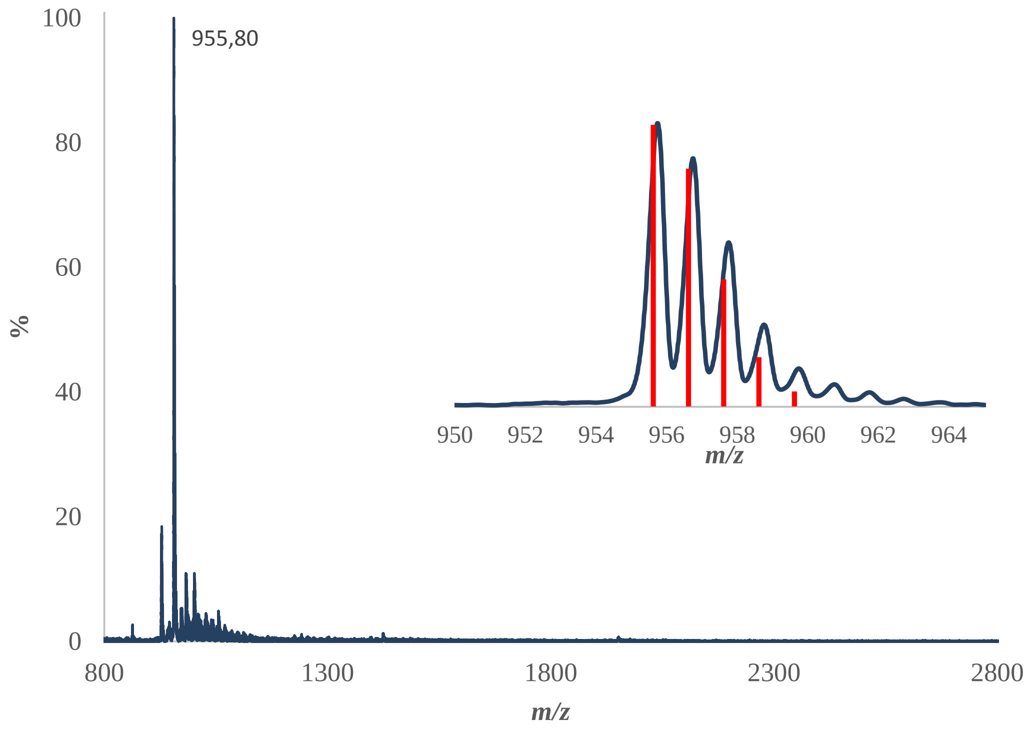

Figure S15. Negative mode ESI-mass spectrum of [K(2,2,2-crypt)][**1**] in THF.

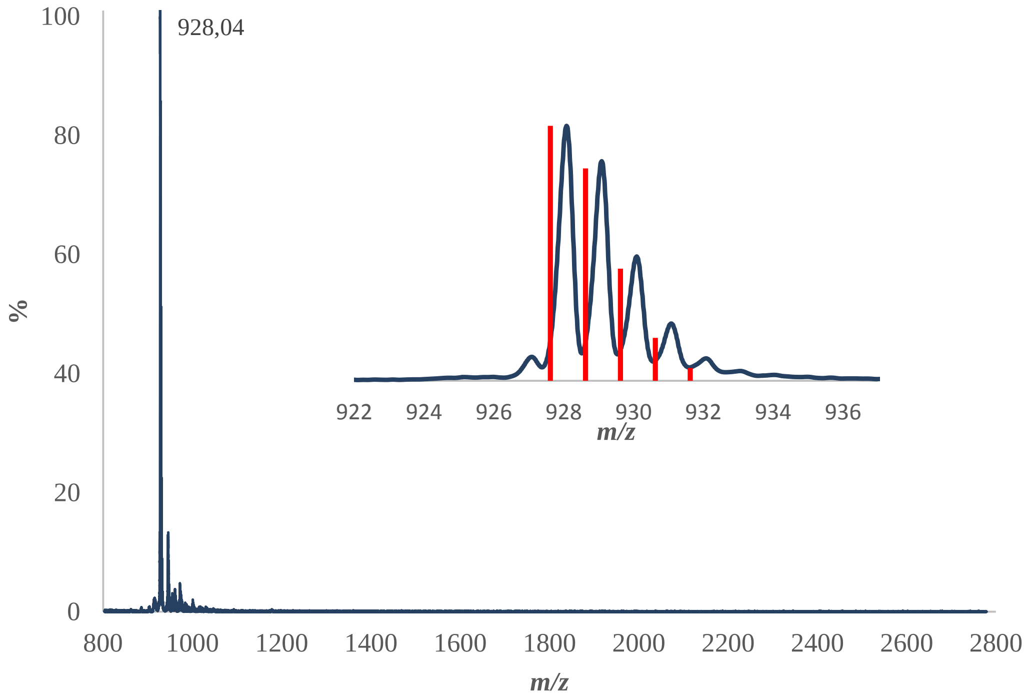

Figure S16. Negative mode ESI-mass spectrum of [K(18-crown-6)][**2**] in THF.

5. UV-Vis spectra

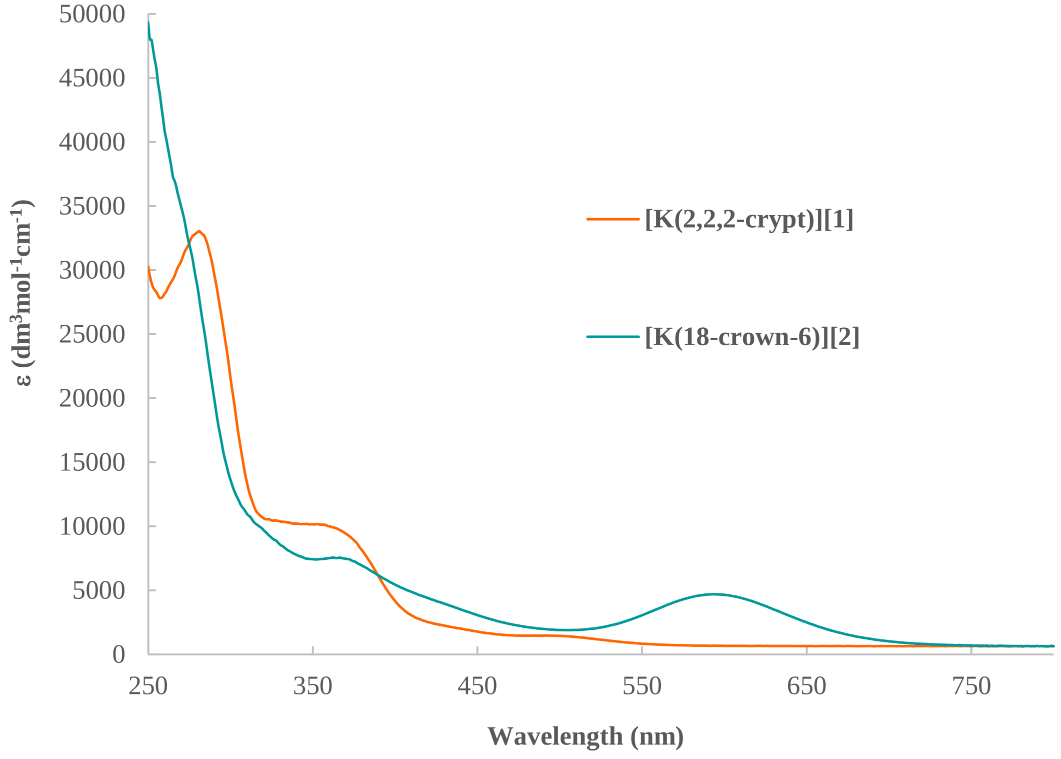

Figure S17. Electronic absorption spectra for [K(2,2,2-crypt)][**1**] (orange) and [K(18-crown-6)][**2**] (blue).

6. IR spectrum

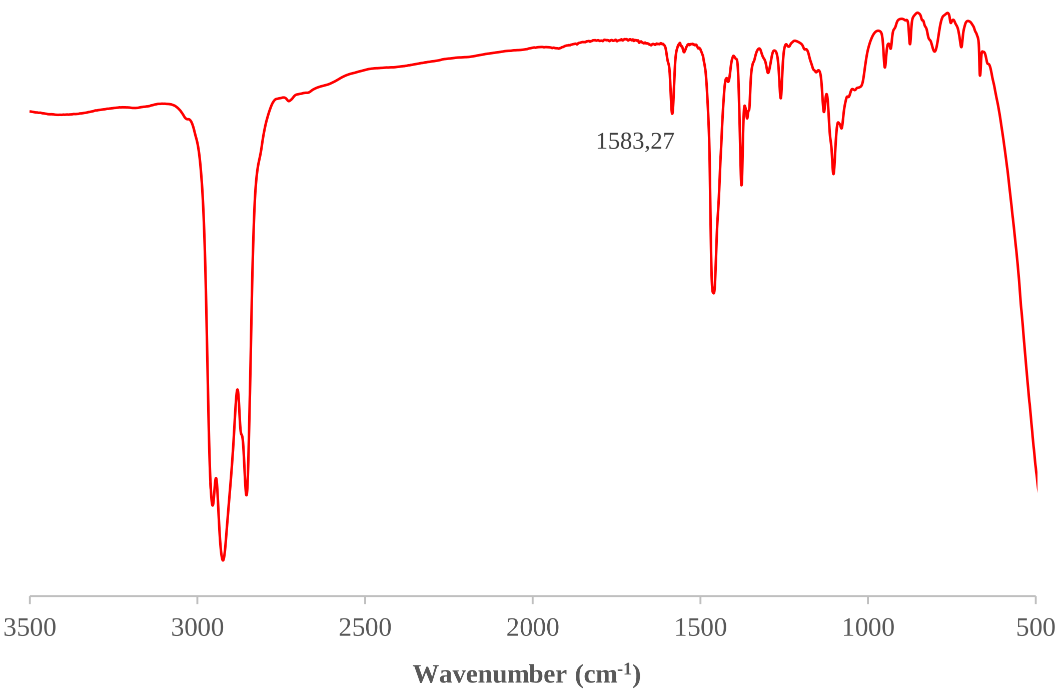

Figure S17. Infrared spectrum of [K(2,2,2-crypt)][**1**] recorded as a Nujol mull.

7. Computational details

Complete reference [22]:

Gaussian 09, Revision D.01, M. J. Frisch, G. W. Trucks, H. B. Schlegel, G. E. Scuseria, M. A. Robb, J. R. Cheeseman, G. Scalmani, V. Barone, B. Mennucci, G. A. Petersson, H. Nakatsuji, M. Caricato, X. Li, H. P. Hratchian, A. F. Izmaylov, J. Bloino, G. Zheng, J. L. Sonnenberg, M. Hada, M. Ehara, K. Toyota, R. Fukuda, J. Hasegawa, M. Ishida, T. Nakajima, Y. Honda, O. Kitao, H. Nakai, T. Vreven, J. A. Montgomery, Jr., J. E. Peralta, F. Ogliaro, M. Bearpark, J. J. Heyd, E. Brothers, K. N. Kudin, V. N. Staroverov, T. Keith, R. Kobayashi, J. Normand, K. Raghavachari, A. Rendell, J. C. Burant, S. S. Iyengar, J. Tomasi, M. Cossi, N. Rega, J. M. Millam, M. Klene, J. E. Knox, J. B. Cross, V. Bakken, C. Adamo, J. Jaramillo, R. Gomperts, R. E. Stratmann, O. Yazyev, A. J. Austin, R. Cammi, C. Pomelli, J. W. Ochterski, R. L. Martin, K. Morokuma, V. G. Zakrzewski, G. A. Voth, P. Salvador, J. J. Dannenberg, S. Dapprich, A. D. Daniels, O. Farkas, J. B. Foresman, J. V. Ortiz, J. Cioslowski, and D. J. Fox, Gaussian, Inc., Wallingford CT, 2013.

1$_{calc}$ at ωB97XD/6-311g(d,p)

Frequencies not calculated

```
Si   0.0192077289    0.0192141221     0.0213898383
Si   -1.6427488617   -1.4365851868    0.8215780658
Si   -1.6378655538   1.1235794961     1.3202390434
C    -2.8761158156   0.0326790213     0.2880103461
P    -1.787990859    -0.5061106979    2.8478689785
O    -3.969853988    0.1241211257     -0.2160711871
C    0.4613895081    -1.9054314771    -6.6465919103
C    2.4006528151    -0.2836971744    -6.5482167344
C    0.937167935     -0.5290483207    -6.1663529789
C    0.7061318515    -0.3643254952    -4.6779713115
C    -0.2023835273   0.5614505881     -4.1924323204
C    1.3614485883    -1.1678321073    -3.7543931225
C    -2.0277551452   -2.5627236512    -3.4632597981
C    -1.1211222766   3.1698558852     -2.8917901364
C    -2.8824932768   1.3772217336     -2.9536700762
C    -0.4534621375   0.7152725916     -2.8287436306
C    -1.4919441112   1.7554247308     -2.431648575
```

C	1.1281009124	-1.0598292674	-2.3872591202
C	2.5179730061	2.3829541908	-2.1444163618
C	-4.1266127604	-2.4152554232	-2.073936695
C	-2.5935466415	-2.4391200588	-2.0459055207
C	0.2295261029	-0.0863597507	-1.8916989715
C	1.5914038587	-3.4909746072	-1.8205148584
C	3.3865839159	-1.7278751104	-1.4905251058
C	1.8827454908	-2.0248420206	-1.4804627623
C	-2.0707805422	-4.8341138175	-1.5590521582
C	1.8091971779	2.4851785728	-0.7880430805
C	-1.5930299857	-7.2994810515	-1.3040200488
C	-2.5174269276	-8.2703579204	-0.5616063502
C	-2.1162745062	-3.5213967514	-1.0886814636
C	-4.221519497	4.3793159099	-0.6688613521
C	1.7693338938	3.9352062767	-0.2923925469
C	-1.685825261	-5.8910098152	-0.7511541957
C	-0.144167224	-7.8013897712	-1.2964830652
C	2.4343294268	1.5294102053	0.2217342953
C	-3.6439010521	3.374693521	0.3284136409
C	3.7352505947	1.8218532932	0.6280981797
C	-1.7743984288	-3.2464082553	0.2518782873
C	1.7719734647	0.3848057452	0.7242602946
C	-1.3698228726	-5.6168566136	0.5753199321
C	-4.7389922506	2.8952309668	1.2917285518
C	4.4127137184	1.0429007119	1.5506767695
C	-1.4128768582	-4.3259894645	1.0919804021
C	-2.4468615396	3.883320986	1.1208135604
C	2.4587070803	-0.4069497553	1.6712999452
C	5.8128992872	1.4028766508	2.003550075
C	6.8133748376	0.2827452348	1.6985294387
C	-1.561165308	2.9717459242	1.7284335587
C	-2.2642699895	5.2504333069	1.3188732483
C	3.7492945255	-0.0616544609	2.0642204576
C	2.6868636062	-2.9054078641	2.0557779922
C	1.8580517691	-1.6435030839	2.3200839187
C	0.2551833653	7.6765170117	1.5556973616
C	-1.1267326007	-4.114794077	2.5718669745
C	-0.5459739867	3.4598481365	2.5787739158
C	-1.241839001	5.7436762704	2.1205579735
C	5.8349284477	1.7767499202	3.4902182673
C	1.8129128675	2.9819027243	3.4820399253
C	-0.4075537514	4.8336368915	2.7580749626
C	-0.1729799892	-5.1447938611	3.1821584857
C	0.3593122633	2.5191164658	3.3621855846
C	-1.0300465353	7.2377731092	2.2691493972
C	-2.4436878979	-4.0843268218	3.3591610314
C	1.6584951174	-1.4335515355	3.8240271397
C	-1.0239038476	7.6843871399	3.7346191373
C	-0.2410206858	2.2691393493	4.7513016624
H	0.5622789139	-1.9982957583	-7.7328791553
H	2.5375680126	-0.3501164693	-7.6324224806
H	0.3294688623	0.2293664662	-6.6738900409
H	-0.5845016063	-2.0712937848	-6.3767615921
H	2.7295180957	0.7043784154	-6.2167538928
H	3.0558304675	-1.0262449616	-6.0826030375
H	1.0520388304	-2.7020009807	-6.1835294253

H	-0.7410959389	1.1825811088	-4.9036827995
H	-1.0982018493	3.2402752066	-3.9842170899
H	2.0651398427	-1.9161952288	-4.1085911349
H	-2.9044348692	1.3905342371	-4.0488957737
H	-2.2596755428	-1.6538440418	-4.02563691
H	-2.4633905377	-3.4080076544	-4.0068221052
H	-0.9420143646	-2.6743017349	-3.4562258588
H	2.0206137844	3.0094646946	-2.8911593844
H	-4.4834056354	-1.6335528621	-2.7514785924
H	-0.1448251468	3.4751411567	-2.5137537685
H	-1.8617706457	3.8860927284	-2.5268654462
H	1.9319809946	-3.7510321675	-2.8282231757
H	-3.6310114264	2.0870630783	-2.5900867476
H	2.5156008515	1.3557268532	-2.5148646696
H	-3.1812316391	0.3873220891	-2.6095335
H	3.8068069426	-1.8775060329	-2.4909071394
H	-4.5129615637	-3.380092992	-2.4226964171
H	3.5580906687	2.7165329174	-2.0631317512
H	-2.3315970087	-5.0364469685	-2.5936165594
H	-1.9251993812	-7.2584603201	-2.347976057
H	-2.2536639476	-1.4754038229	-1.6635010176
H	0.5224264135	-3.7043998877	-1.7522950036
H	-3.5510719926	-7.9162090745	-0.5813194782
H	-1.5415106685	1.7739766179	-1.3408757378
H	-4.9639341978	3.8818231001	-1.2990456847
H	-4.5315734015	-2.2010786622	-1.084698928
H	3.5855079575	-0.6981396426	-1.1842064181
H	-2.4824546813	-9.2655813352	-1.0167385104
H	1.2953322977	4.5739953134	-1.0446348176
H	2.1083345681	-4.1500597134	-1.1154964653
H	-3.4489545222	4.798230766	-1.3194936878
H	0.7690440892	2.1946607309	-0.9315971274
H	3.9179050619	-2.3905673381	-0.8007671512
H	-0.0720527061	-8.7976224062	-1.745259143
H	1.5268806906	-1.8826065067	-0.4598248134
H	-4.7262722076	5.2099783114	-0.1638219836
H	2.7720906247	4.3337583998	-0.1076434439
H	0.5048880901	-7.1211674136	-1.8535582936
H	4.2337510743	2.6976306022	0.2212212696
H	-3.3317916731	2.4988777263	-0.245539731
H	-2.2195479887	-8.3716195142	0.4865592142
H	1.1874946917	4.0229553611	0.6264562208
H	-5.5803905261	2.4798878929	0.7318888586
H	0.2392746545	-7.8637712882	-0.2733529502
H	6.1200202687	2.2879926523	1.4339052022
H	2.7562585174	-3.1261370633	0.9888373255
H	6.8067735778	0.0340723948	0.6343266121
H	-5.0937173714	3.7300644975	1.9069401283
H	-1.0763496339	-6.4357886584	1.2234652648
H	-2.9312025475	5.9589995016	0.8379698006
H	-4.3670806855	2.1116182542	1.9555941208
H	0.8673335656	-1.7963372703	1.8888297564
H	7.8289528573	0.5786477182	1.9810647106
H	0.2343258991	7.383561067	0.5031373648
H	3.7055890618	-2.8086675101	2.4449946857
H	2.2413977957	3.2227601569	2.5081994944

H	1.1293787115	7.2040819582	2.0143829547
H	2.2257622264	-3.7646739447	2.5482108187
H	4.2525699533	-0.680618092	2.801663119
H	6.5627810077	-0.6268074259	2.2531355822
H	5.1331670724	2.5885292799	3.6971504491
H	0.7110034353	-5.2978105511	2.5580598533
H	-0.6623104129	-3.1316704998	2.6887806408
H	0.3779077659	1.5563291476	2.8452541959
H	-1.8729995021	7.734315543	1.774179832
H	6.8361732751	2.0935351558	3.800273538
H	-3.1106879207	-3.3077127858	2.981290798
H	0.3849766278	8.7623760918	1.6136451637
H	-0.6586386182	-6.1162903993	3.3247989991
H	2.4141217565	2.1758294945	3.9127906711
H	-2.9525990665	-5.0521150051	3.282111598
H	5.5452393645	0.9219732611	4.1092340778
H	1.9148010404	3.8563938887	4.1344938006
H	0.3853150409	5.20155554	3.402043324
H	1.0378097021	-0.5554365091	4.0071059232
H	2.6190769416	-1.3176313679	4.339376073
H	1.1378526873	-2.293553034	4.2573357727
H	0.1583027092	-4.8006510199	4.1662158636
H	-2.2532427511	-3.8709697333	4.4152996644
H	-1.9367163393	7.3677196227	4.2451596203
H	-1.2583004972	1.8804253543	4.674492216
H	-0.9458574108	8.7740147453	3.807418094
H	0.3572655536	1.5337691147	5.2982137362
H	-0.1743095417	7.2543402336	4.2732015048
H	-0.2630825919	3.1997684454	5.330281777

Excerpt from TDDFT output for **1**$_{calc}$

```
Excited State   1:      Singlet-A      2.8330 eV  437.64 nm  f=0.0033
<S**2>=0.000
     260 -> 264         0.18852
     261 -> 264         0.54062
     261 -> 265         0.20193
     261 -> 270         0.16270
     261 -> 273        -0.13460
     262 -> 264        -0.15025
 This state for optimization and/or second-order correction.
 Total Energy, E(TD-HF/TD-KS) =  -3665.02370041
 Copying the excited state density for this state as the 1-particle RhoCI
density.

 Excited State   2:      Singlet-A      3.0498 eV  406.53 nm  f=0.0101
<S**2>=0.000
     261 -> 264         0.14980
     262 -> 264         0.59997
     262 -> 265         0.22267
     262 -> 270         0.17131

 Excited State   3:      Singlet-A      3.8467 eV  322.31 nm  f=0.0046
<S**2>=0.000
     262 -> 263        -0.28065
     262 -> 265         0.36476
     262 -> 269        -0.11378
     262 -> 270        -0.10641
     262 -> 271        -0.21894
     262 -> 272         0.34292
     262 -> 274         0.14618
     262 -> 277         0.18194

 Excited State   4:      Singlet-A      3.8982 eV  318.05 nm  f=0.0031
<S**2>=0.000
     262 -> 263         0.60491
     262 -> 264        -0.11238
     262 -> 265         0.23110
     262 -> 272         0.12273

 Excited State   5:      Singlet-A      4.0587 eV  305.47 nm  f=0.0014
<S**2>=0.000
     262 -> 264        -0.19793
     262 -> 265         0.44546
     262 -> 266        -0.13818
     262 -> 269         0.14931
     262 -> 270         0.13168
     262 -> 271         0.18134
     262 -> 272        -0.25343
     262 -> 274        -0.15512
     262 -> 286         0.11268

 Excited State   6:      Singlet-A      4.2390 eV  292.48 nm  f=0.2446
<S**2>=0.000
     260 -> 264        -0.13867
     261 -> 263         0.62372
     261 -> 264         0.13717
```

GIAO calculations for **1**$_{calc}$

References at: $SiMe_4$ (^{29}Si : 355.4917, ^{13}C: 190.2308);

PMe_3 (^{31}P: 391.7033)

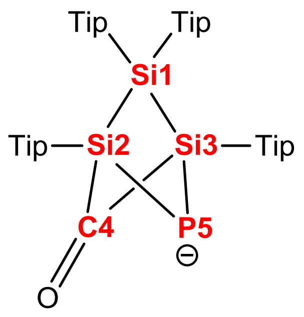

**Atom**	**δ_{exp} ([D_8]-THF)**	**δ_{calc} (scrf(cpcm,solvent=THF))**
Si1	−1.8 ppm	+2.0 ppm
Si2	−10.9 ppm	−9.1 ppm
Si3	− 16.1 ppm	−4.4 ppm
C4	+222.7 ppm	+251.9 ppm
P5	+322.1 ppm	+330.4 ppm (vs. H_3PO_4, 85%)

Excerpt from Gaussian09/GIAO output

```
   1  Si    Isotropic =   353.5305   Anisotropy =    88.8292
XX=   351.0301   YX=   -63.0122   ZX=    31.1742
XY=   -76.2070   YY=   329.1385   ZY=    61.0599
XZ=   -14.0544   YZ=   -66.9644   ZZ=   380.4227
Eigenvalues:   269.5196   378.3219   412.7499
   2  Si    Isotropic =   364.5824   Anisotropy =    91.7391
XX=   398.3232   YX=    19.5724   ZX=   -18.0476
XY=   -51.1168   YY=   343.2255   ZY=   -63.6526
XZ=   -65.7642   YZ=   -37.3746   ZZ=   352.1984
Eigenvalues:   282.7670   385.2384   425.7418
   3  Si    Isotropic =   359.8810   Anisotropy =   126.0777
XX=   421.9073   YX=   -61.4136   ZX=    26.7993
XY=    47.8832   YY=   323.0199   ZY=   -16.6251
XZ=    67.5103   YZ=   -17.0764   ZZ=   334.7156
Eigenvalues:   304.6486   331.0615   443.9328
   4  C     Isotropic =   -61.6916   Anisotropy =   127.4301
XX=  -108.3243   YX=     6.6741   ZX=    -7.0545
XY=    -1.9012   YY=   -96.5810   ZY=   -77.5878
XZ=     3.0552   YZ=    37.2900   ZZ=    19.8304
Eigenvalues:  -108.8281   -99.5085    23.2618
   5  P     Isotropic =   661.9534   Anisotropy =   330.2040
XX=   822.5360   YX=   -98.3477   ZX=    70.7718
XY=  -146.2944   YY=   614.8321   ZY=   281.1819
XZ=    65.0030   YZ=   125.8007   ZZ=   548.4921
Eigenvalues:   339.5760   764.1948   882.0894
   6  O     Isotropic =  -225.2735   Anisotropy =   858.8743
XX=  -251.6042   YX=   -50.7408   ZX=   -55.2200
XY=   -80.2073   YY=  -734.1546   ZY=  -385.1482
XZ=   -13.2991   YZ=   -12.4324   ZZ=   309.9384
Eigenvalues:  -780.1581  -242.9717   347.3094
```

2$_{calc}$ at ωB97XD/6-311g(d,p)

Frequencies not calculated

Charge = −1   Multiplicity = 1

P	-1.5833448362	-0.6831977271	-2.1173348066
Si	0.4461632605	-0.2811692734	-1.1720164228
Si	-2.1401138099	-0.6004479537	-0.063217246
Si	-0.2223346992	-0.9900681206	0.9995722828
C	4.4136984231	-3.5574041734	-6.0332021339
C	0.772811665	0.1326350014	-5.4369983858
C	4.8699657098	-3.1580661962	-4.6258841105
C	6.0937495952	-2.2360229511	-4.6831566052
C	2.4976251963	1.6048910015	-4.2937059973
C	3.1098483731	-1.3767502793	-4.2489323821
C	1.4762978942	0.4749942583	-4.1189620019
C	3.7510208715	-2.5273633813	-3.8211917779
C	-0.7840921015	3.3441579002	-3.5963698748
C	2.1109695012	-0.7610430352	-3.4958767348
C	3.3359228168	-3.0778733607	-2.6184192971
C	3.6500733162	5.8300992518	-2.3945651519
C	1.7286551351	-1.2841789801	-2.2397110232
C	-0.7502410122	2.7714481625	-2.1751020649
C	2.3436157136	-2.4933903047	-1.8335081412
C	-4.8808160565	1.6083391987	-1.6244375871
C	1.3654089544	3.8851829537	-1.5435148706
C	0.6545205519	2.6894005279	-1.5888340506
C	3.3957310503	5.2887529912	-0.9832193352
C	-1.6836021114	3.5755041589	-1.2658127797
C	2.6463340353	3.972682328	-1.020709896
C	1.2353044413	1.4932907053	-1.097183768
C	4.4792876232	-0.0013147102	-0.7754051221
C	0.6509629871	-4.043247349	-0.8375998694
C	3.2038854871	2.8122891378	-0.5120347767
C	2.5284404443	1.5909903401	-0.5270361908
C	1.9171502336	-3.2211283894	-0.5719619782
C	2.6657830168	6.3220385163	-0.1172349932
C	-4.4715622639	1.5423452217	-0.1483480464
C	2.9980431299	-4.0816114032	0.0819328663
C	3.276631478	0.4085539946	0.084513014
C	-3.6568066462	-4.1884509427	-0.2416661947
C	-4.8632368635	0.1968626847	0.439402238
C	-8.751154137	-2.5229228405	1.005148534
C	-3.9367691568	-0.858091033	0.4979395612
C	-5.0202737929	2.7414750527	0.6279485247
C	-6.1751651046	-0.0075718268	0.8637006312
C	-4.3620376129	-2.1149445914	0.978054709
C	-6.6023446557	-1.2384186091	1.3423030521
C	3.7225038539	0.6870824183	1.5266316496
C	-5.6821837966	-2.2808711198	1.3863049963
C	-8.0319717858	-1.4345288179	1.8094417473
C	-3.4236383434	-3.3127335787	0.9948200059
C	0.2719722297	3.5713402175	1.8270655965
C	-0.4360633597	2.2238437207	1.9902840354

C	-1.8706712812	2.4043413393	2.4972440542
C	-3.5027480803	-4.1309572801	2.2856499719
C	0.4537169151	-0.1118068503	2.5760367745
C	-8.0930280703	-1.7300764051	3.3127450548
C	0.3203575598	1.258491379	2.8852743639
C	1.3575688818	-2.4229179826	3.2649085767
C	2.7394970911	-2.9497508128	3.6581131362
C	1.1820075956	-0.9232810969	3.4834041785
C	0.2433144809	-3.1976119156	3.9756271425
C	0.8815533766	1.76873679	4.0573097305
C	1.7379654918	-0.3699351091	4.6324392744
C	1.6011560285	0.9779359782	4.9379063002
C	2.2623546369	1.5761808759	6.1640313822
C	3.7877024043	1.5875585464	6.0021016676
C	1.8519126464	0.8613956261	7.455502659
H	4.1256895176	-2.6769159519	-6.6156913805
H	5.2178571187	-4.0671365381	-6.5741805608
H	1.4800973795	-0.2186173711	-6.1956357805
H	3.5488041043	-4.2236680377	-5.9877504329
H	0.2636749208	1.0157559842	-5.837042406
H	6.9259700775	-2.7184703321	-5.2067038014
H	3.3952364077	-0.9345178859	-5.1996263152
H	3.2878511837	1.3171992538	-4.9951919799
H	0.0238391825	-0.6464512901	-5.2760691226
H	5.8535597211	-1.3087912043	-5.212733768
H	2.0169125337	2.506260046	-4.6856728391
H	5.1672356921	-4.073461395	-4.1004958964
H	-0.1312217373	2.7851439349	-4.2681439765
H	-1.8022056577	3.2820599334	-3.9935306715
H	6.423368947	-1.9665775533	-3.676698286
H	-0.4778788208	4.3955450653	-3.6263411347
H	2.962420165	1.8617899268	-3.3389748561
H	0.6967384267	0.8360498608	-3.4524238862
H	4.194334154	5.0995335202	-2.9983277794
H	2.7057689356	6.0446194051	-2.9044879052
H	4.2326401129	6.7567675342	-2.3599443976
H	3.8113949637	-3.9934602837	-2.2823919684
H	-4.3629469021	0.8413980914	-2.2048801904
H	-1.1572751011	1.7625285618	-2.230425258
H	-4.6343907038	2.5846622491	-2.053261026
H	0.8928247005	4.7838053074	-1.9308908522
H	4.1836200279	-0.223451908	-1.8017674805
H	-5.9605292377	1.4544210435	-1.7253447354
H	-2.6892411127	3.6144778762	-1.6945541403
H	0.8565935952	-4.8358213776	-1.5665264794
H	-1.3326376209	4.6053477731	-1.1349274875
H	4.3701169095	5.0943101059	-0.519453357
H	5.2264631819	0.7998732661	-0.7984433553
H	-0.1501181126	-3.4122491493	-1.2305280211
H	3.2393517726	-4.9727344097	-0.5073305054
H	1.6886061609	6.5668436086	-0.5449672756
H	4.9561437822	-0.894924844	-0.359798654
H	3.2429810419	7.2498376058	-0.0446279222
H	-3.5143017381	-3.607516711	-1.1562677474
H	-8.714059433	-2.306058887	-0.0652451053
H	4.201260911	2.8604117809	-0.0835323244

H	-1.7563703024	3.1179225248	-0.2786756334
H	-3.3780514231	1.6128336244	-0.1076964438
H	3.9208874147	-3.5161660472	0.2427480341
H	2.5994092076	-0.4452882384	0.1324198592
H	-4.6739211713	-4.5970948506	-0.2452823219
H	1.6334511557	-2.4617118068	0.1584915382
H	0.2916473499	-4.5008637054	0.0901187567
H	-4.5549241772	3.6623001451	0.2632374577
H	-2.9495517489	-5.0235462084	-0.2577570937
H	-6.8880724644	0.8110501185	0.8175383328
H	2.4978999446	5.9359090608	0.8912752796
H	-6.1021876676	2.847036322	0.4980758238
H	-9.8007245309	-2.6008125384	1.3069565187
H	-8.2844511859	-3.4999518737	1.1630502858
H	2.6405655091	-4.4287568109	1.0555986112
H	-8.559707388	-0.4893535698	1.6355110728
H	-0.4905932183	1.7606749764	1.0033862305
H	4.5237096499	1.43284038	1.5622138198
H	-0.2251766345	4.1676199365	1.059250111
H	-4.811339464	2.6527003664	1.6966689367
H	-2.399177646	-2.9351316169	0.9330660404
H	1.3085594577	3.4284221147	1.5131486238
H	-6.0033642338	-3.2537805254	1.7472893323
H	4.1082998094	-0.2305502341	1.9818733773
H	2.8948110458	1.0423645944	2.1415180639
H	-2.4177269003	3.1166121025	1.8712943536
H	1.2291930318	-2.6134748033	2.195898822
H	-4.4582702315	-4.6575204468	2.3842200521
H	-2.4119070588	1.4534124969	2.4831572488
H	0.2647429704	4.1585194429	2.7517163822
H	-2.7103840828	-4.8853260669	2.2958961797
H	-9.1308849574	-1.8099666571	3.6525213056
H	-7.5887626909	-2.6735323502	3.5434159385
H	3.5332824938	-2.3622223879	3.188733644
H	2.8477841803	-3.9882510997	3.3316163601
H	-3.3735550813	-3.4931988531	3.1641967577
H	-7.6001151044	-0.9405168527	3.8852557204
H	-1.8765345671	2.7846789459	3.5246915805
H	0.3565997347	-4.275536343	3.8167806579
H	-0.7336497341	-2.8975847698	3.5871011221
H	2.899450469	-2.9353886684	4.7412036796
H	0.7701701751	2.8246798164	4.285364051
H	0.2603411429	-3.0033155791	5.0536894346
H	2.3032443539	-1.0048946644	5.3070031724
H	4.0775714328	2.1307539964	5.0992716801
H	1.9268549708	2.6172990413	6.2389863787
H	4.1702249456	0.5661661197	5.9113600052
H	4.2730574108	2.0579992662	6.8638231033
H	0.765477908	0.8585212702	7.5736381371
H	2.1901362364	-0.1792186548	7.4535487403
H	2.2945976948	1.3530410782	8.3279278463

Excerpt from TDDFT output for **2**$_{calc}$

```
Excited State   1:      Singlet-A      2.4267 eV  510.92 nm
f=0.1196  <S**2>=0.000
     255 -> 256        0.68723
 This state for optimization and/or second-order correction.
 Total Energy, E(TD-HF/TD-KS) =  -3551.66480125
 Copying the excited state density for this state as the 1-particle
RhoCI density.

 Excited State   2:      Singlet-A      2.9968 eV  413.72 nm
f=0.0115  <S**2>=0.000
     255 -> 257        0.63943
     255 -> 258       -0.17386

 Excited State   3:      Singlet-A      3.4859 eV  355.67 nm
f=0.0125  <S**2>=0.000
     254 -> 256        0.13961
     255 -> 257        0.15739
     255 -> 258        0.61643
     255 -> 259       -0.10790
     255 -> 269        0.10318

 Excited State   4:      Singlet-A      3.6913 eV  335.88 nm
f=0.0819  <S**2>=0.000
     254 -> 256        0.65236
     254 -> 258        0.10775
     255 -> 258       -0.12688

 Excited State   5:      Singlet-A      3.9237 eV  315.99 nm
f=0.0582  <S**2>=0.000
     255 -> 259        0.52354
     255 -> 261        0.19289
     255 -> 262        0.34801

 Excited State   6:      Singlet-A      3.9771 eV  311.75 nm
f=0.0590  <S**2>=0.000
     253 -> 256       -0.33661
     255 -> 259       -0.27758
     255 -> 261        0.38059
     255 -> 262        0.27573
     255 -> 264        0.12666
```

GIAO calculations for **2calc**

Reference at: $SiMe_4$ (^{29}Si : 355.4917); PMe_3 (^{31}P: 391.7033, corrected to H_3PO_4 reference by subtracting 61 ppm)

**Atom**	**δ_{exp} ([D_8]-THF)**	**δ_{calc} (scrf(cpcm,solvent=THF))**
P1	−55.7 ppm	−38.5 ppm (vs. H_3PO_4, 85%)
Si2	−14.9 ppm	−11.5 ppm
Si3	+193.1 ppm	+283.8 ppm
Si4	−39.9 ppm	−43.4 ppm

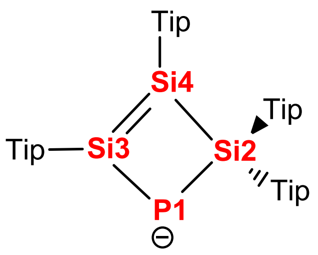

Excerpt from Gaussian09/GIAO output

```
     1  P    Isotropic =   369.2374   Anisotropy =   332.7304
   XX=   189.0080   YX=   109.4139   ZX=    58.4166
   XY=   166.0949   YY=   421.6812   ZY=   -51.3290
   XZ=   124.1234   YZ=   178.0732   ZZ=   497.0230
   Eigenvalues:   116.1991   400.4555   591.0576
     2  Si   Isotropic =   367.0110   Anisotropy =   125.5141
   XX=   341.3840   YX=   -23.2226   ZX=    -6.4213
   XY=   -29.8653   YY=   312.7613   ZY=   -43.6884
   XZ=   -10.1009   YZ=    -1.1877   ZZ=   446.8876
   Eigenvalues:   293.3131   357.0327   450.6870
     3  Si   Isotropic =    71.7250   Anisotropy =   447.5116
   XX=  -192.2314   YX=    31.4201   ZX=    -2.9453
   XY=   -10.1350   YY=    60.2929   ZY=    80.7149
   XZ=    68.4147   YZ=    79.4837   ZZ=   347.1134
   Eigenvalues:  -194.3490    39.4578   370.0660
     4  Si   Isotropic =   398.9373   Anisotropy =   113.0151
   XX=   388.2144   YX=   -52.6351   ZX=    24.1307
   XY=   -20.8783   YY=   458.2449   ZY=   -20.3471
   XZ=   -28.1641   YZ=    31.5645   ZZ=   350.3525
   Eigenvalues:   350.0613   372.4699   474.2807
```

[K(18-crown-6)][**2** calc] at ωB97XD/6-311g(d,p)

Frequencies not calculated

Charge = −1 Multiplicity = 1 (atom list rearranged)

P	1.04963	-0.58766	0.41803
Si	-1.17927	-0.82424	-0.0141
Si	0.54146	1.47678	0.0992
Si	-1.36485	1.32132	-0.98664
K	4.10316	-1.48513	-0.03781
O	5.38217	-0.39406	2.13416
O	5.9549	0.76564	-0.35406
O	4.63535	-3.09017	2.34008
O	4.40156	-0.24829	-2.44378
O	3.27594	-4.08785	0.10646
O	3.2587	-2.82734	-2.39944
C	1.52479	3.4969	3.82653
C	-5.97018	-0.28861	5.02964
C	-6.62291	5.94584	-1.37064
C	-1.90055	3.66594	2.18388
C	-3.78527	2.14091	2.8932
C	-6.67401	4.60952	-0.62292
C	-4.67056	3.52972	0.47032
C	-0.7111	0.40618	4.103
C	-7.81972	3.73038	-1.13857
C	-2.80586	2.49929	1.77431
C	4.50294	7.18367	0.42339
C	-5.73122	-1.59732	4.266
C	1.92804	2.47328	2.76162
C	-5.35287	3.86949	-0.68742
C	-5.48476	-2.7622	5.22963
C	3.46199	4.06676	1.57782
C	5.05169	5.76105	0.58775
C	2.94186	1.46731	3.30785
C	-3.48016	2.80256	0.44793
C	-3.29049	-1.27587	3.67508
C	2.42521	3.13663	1.48704
C	3.93823	4.7393	0.46305
C	-4.60629	-1.43072	3.26519
C	-0.85689	-0.93889	3.38739
C	6.19739	5.49893	-0.39516
C	-4.80041	3.48677	-1.9033
C	-0.49072	-2.08851	4.33329
C	-2.24422	-1.08825	2.77737
C	-2.92869	2.40304	-0.78812
C	-4.85678	-1.37348	1.90488
C	1.84779	2.85092	0.23747
C	3.32088	4.49446	-0.76175
C	-3.61046	2.77083	-1.97441
C	2.27912	3.58267	-0.89539
C	-2.49866	-1.03718	1.38521
C	-3.84447	-1.16969	0.96674
C	-5.40521	-0.13502	-0.75877
C	6.31617	0.65228	1.99808

C	5.52761	-1.12235	3.32997
C	5.91505	1.52152	0.83681
C	1.54976	4.661	-3.10041
C	1.62351	3.38749	-2.25712
C	-4.27961	-1.14402	-0.49542
C	4.45088	-2.17319	3.39759
C	-2.13151	3.59092	-3.82017
C	-3.04322	2.45108	-3.35255
C	-4.10041	2.12848	-4.40967
C	-2.24547	-4.40794	1.67859
C	-4.68654	-2.54364	-0.97332
C	-0.9591	-3.82799	1.08129
C	5.59596	1.54594	-1.47704
C	2.29257	2.25572	-3.04277
C	0.26578	-4.64332	1.49724
C	-1.27196	-2.44184	-1.08223
C	3.74225	-4.18184	2.42224
C	-1.07587	-3.68602	-0.43186
C	5.45916	0.64968	-2.6801
C	-1.7763	-1.23483	-3.31283
C	-1.44073	-2.46923	-2.48639
C	-0.53811	-0.63339	-3.97917
C	3.74904	-4.94311	1.11912
C	-1.02474	-4.86876	-1.16455
C	-2.88887	-1.47719	-4.33751
C	-1.33591	-3.675	-3.18352
C	3.90066	-0.88254	-3.60078
C	-1.13057	-4.88798	-2.548
C	2.75643	-1.77216	-3.19289
C	2.22772	-3.70241	-1.97767
C	2.82189	-4.74001	-1.06398
C	-2.26446	-7.09002	-2.97474
C	-1.07441	-6.18896	-3.32525
C	0.25178	-6.92832	-3.1162
H	-6.80928	-0.38783	5.72514
H	-7.56784	6.48605	-1.25889
H	-2.48076	4.58777	2.29585
H	1.02727	2.99347	4.66076
H	2.39456	4.02524	4.22924
H	-5.08249	-0.01154	5.60725
H	-6.87024	4.82611	0.43338
H	0.8368	4.23955	3.41703
H	-4.36901	3.00418	3.22889
H	-5.81681	6.57805	-0.99031
H	-1.41086	3.4577	3.14083
H	-6.18554	0.53178	4.34079
H	-5.09359	3.83071	1.42343
H	-3.23833	1.76203	3.75958
H	3.71537	7.38388	1.15356
H	-8.78265	4.23729	-1.0235
H	-1.42318	0.49739	4.93021
H	-1.12481	3.84296	1.43266
H	5.29516	7.92642	0.55678
H	5.45871	5.67481	1.60204
H	-6.45182	5.79191	-2.44037
H	0.29788	0.51562	4.51335

H	3.90739	4.27746	2.54689
H	-0.88863	1.23425	3.41481
H	-6.34183	-2.90038	5.8955
H	-4.47372	1.35691	2.57036
H	2.56076	0.99352	4.21812
H	-4.60618	-2.57785	5.85521
H	-7.85956	2.78297	-0.5954
H	-3.06699	-1.29586	4.73783
H	3.88878	1.96173	3.55596
H	-6.64095	-1.82243	3.69742
H	1.02598	1.90684	2.50967
H	4.07493	7.31981	-0.57444
H	-2.17014	1.62766	1.61388
H	-7.68448	3.50054	-2.19997
H	-1.07316	-2.06573	5.25986
H	7.0097	6.21424	-0.23823
H	-5.3175	-3.69456	4.6846
H	0.56692	-2.01828	4.6099
H	3.14433	0.68623	2.5735
H	6.60174	4.49056	-0.27258
H	-5.31751	3.74947	-2.82002
H	-0.1247	-0.94358	2.58032
H	6.32798	1.27147	2.90696
H	-5.88068	-1.48846	1.56054
H	-5.16532	0.8477	-0.35079
H	5.42502	-0.45167	4.19561
H	5.86301	5.60195	-1.43167
H	3.65253	5.04768	-1.63423
H	-0.65517	-3.05977	3.86232
H	-6.35276	-0.46712	-0.3231
H	1.1181	5.49057	-2.53478
H	4.90784	1.92518	0.99778
H	6.61248	2.3703	0.77632
H	-2.70088	4.51888	-3.93624
H	-1.33848	3.77148	-3.0906
H	3.45956	-1.70321	3.32153
H	7.32365	0.24104	1.84424
H	-5.56104	-0.02326	-1.83612
H	4.51819	-2.68473	4.36851
H	0.59656	3.06481	-2.0728
H	6.51777	-1.59737	3.37093
H	2.53213	4.97305	-3.47009
H	-2.16963	-4.47377	2.76834
H	-4.68588	3.00876	-4.69246
H	0.91797	4.48343	-3.97492
H	-3.43418	-0.82133	-1.10354
H	-0.81144	-2.83986	1.50919
H	-3.10699	-3.78099	1.44263
H	-2.40799	1.56626	-3.24674
H	-5.5404	-2.91348	-0.39652
H	-4.79318	1.35898	-4.05849
H	4.64495	2.06052	-1.29084
H	-1.66598	3.35013	-4.78185
H	6.36557	2.30739	-1.6721
H	0.36893	-4.63998	2.58724
H	2.34844	1.33843	-2.45186

H	3.31104	2.54134	-3.33141
H	2.72628	-3.81951	2.61985
H	-2.43265	-5.41393	1.28817
H	-3.86716	-3.25757	-0.87023
H	-4.98204	-2.51661	-2.02664
H	-3.61574	1.76272	-5.32003
H	-2.14415	-0.47548	-2.62331
H	4.03854	-4.85412	3.24055
H	1.16332	-4.20216	1.06346
H	1.72673	2.03985	-3.95429
H	0.1679	-0.28641	-3.22206
H	0.19793	-5.68996	1.18281
H	-0.81592	0.23299	-4.58891
H	5.24379	1.27366	-3.55839
H	6.39439	0.10317	-2.86653
H	-3.21498	-0.52268	-4.75811
H	-3.75429	-1.95835	-3.87564
H	-0.90801	-5.80933	-0.63424
H	3.08745	-5.81395	1.22665
H	4.76017	-5.30217	0.88226
H	2.01951	-1.19479	-2.61965
H	-0.04662	-1.36678	-4.62835
H	1.44301	-3.1441	-1.45139
H	-2.55987	-2.10323	-5.17253
H	3.53463	-0.12898	-4.31241
H	-1.44787	-3.67184	-4.26371
H	2.04343	-5.47255	-0.81153
H	-2.23027	-7.38865	-1.92249
H	4.68967	-1.47403	-4.08583
H	-3.21003	-6.56907	-3.14181
H	2.26309	-2.16888	-4.09111
H	3.65474	-5.25949	-1.55793
H	1.76637	-4.19515	-2.84329
H	-1.15052	-5.93622	-4.38901
H	0.39144	-7.19407	-2.06351
H	-2.25788	-7.99984	-3.58272
H	1.10155	-6.31258	-3.42295
H	0.277	-7.85408	-3.69848

Excerpt from TDDFT output for [K(18-crown-6)][**2**]$_{calc}$

```
Excited State   1:      Singlet-A      2.4101 eV  514.44 nm
f=0.1304  <S**2>=0.000
     336 -> 337        0.68629
 This state for optimization and/or second-order correction.
 Total Energy, E(TD-HF/TD-KS) =  -5074.62431783
 Copying the excited state density for this state as the 1-particle
RhoCI density.

 Excited State   2:      Singlet-A      3.0414 eV  407.65 nm
f=0.0037  <S**2>=0.000
     336 -> 339        0.65200
     336 -> 340       -0.12124
     336 -> 350        0.12637

 Excited State   3:      Singlet-A      3.5538 eV  348.88 nm
f=0.0156  <S**2>=0.000
     336 -> 339        0.13423
     336 -> 340        0.62047
     336 -> 344        0.11696
     336 -> 351        0.13581

 Excited State   4:      Singlet-A      3.7559 eV  330.11 nm
f=0.0988  <S**2>=0.000
     335 -> 337        0.65314

 Excited State   5:      Singlet-A      4.0006 eV  309.92 nm
f=0.0987  <S**2>=0.000
     325 -> 337        0.10180
     332 -> 337       -0.10220
     334 -> 337        0.47283
     336 -> 342       -0.19518
     336 -> 343        0.12893
     336 -> 344       -0.16168
     336 -> 347        0.11439
     336 -> 348       -0.10790
     336 -> 350       -0.25841

 Excited State   6:      Singlet-A      4.0128 eV  308.97 nm
f=0.0231  <S**2>=0.000
     334 -> 337        0.41349
     336 -> 342        0.22947
     336 -> 343       -0.16634
     336 -> 344        0.29311
     336 -> 350        0.26242
```

Selected Molecular Orbitals of [K(18-crown-6)][**2**]$_{calc}$

MO 326 (isovalue 0.05)

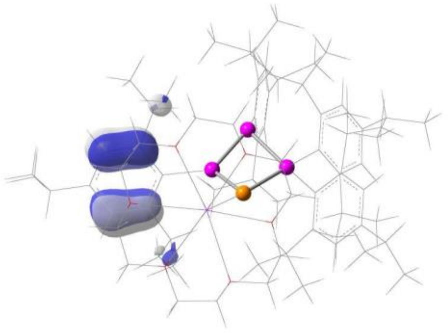

MO 327 (isovalue 0.05)

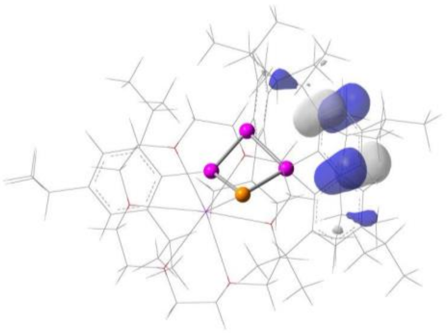

MO 328 (isovalue 0.05)

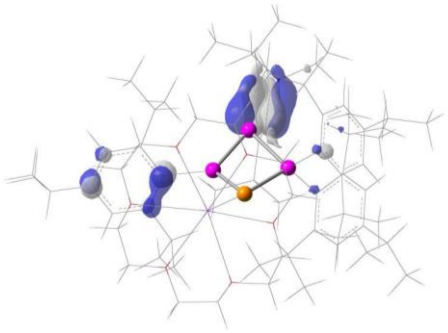

MO 329 (isovalue 0.05)

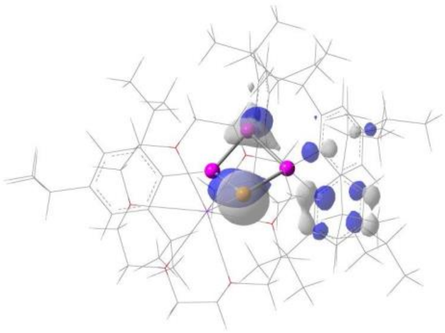

MO 330 (isovalue 0.05)

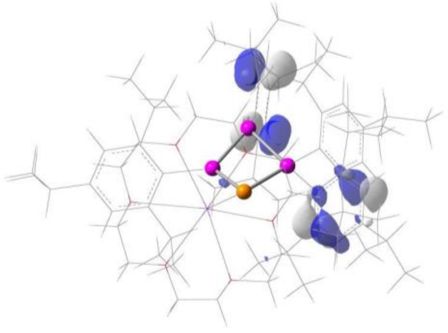

MO 331 (isovalue 0.05)

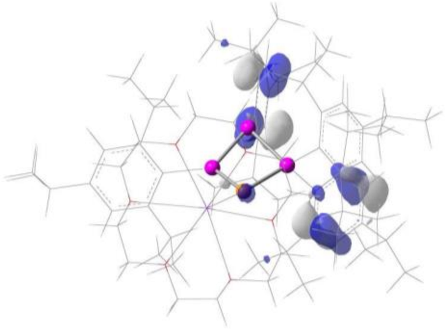

MO 332 (isovalue 0.05)

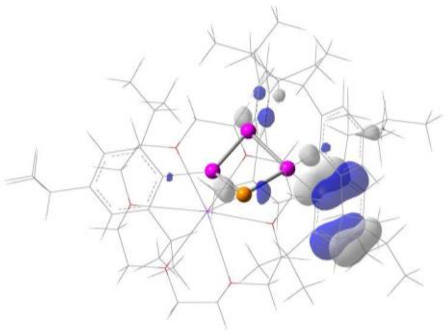

MO 333 (isovalue 0.05)

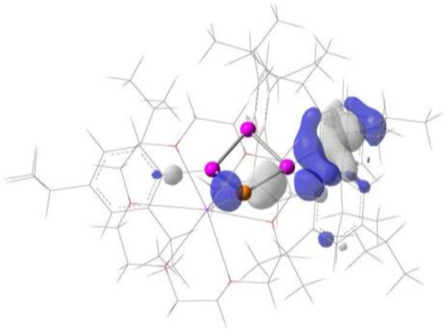

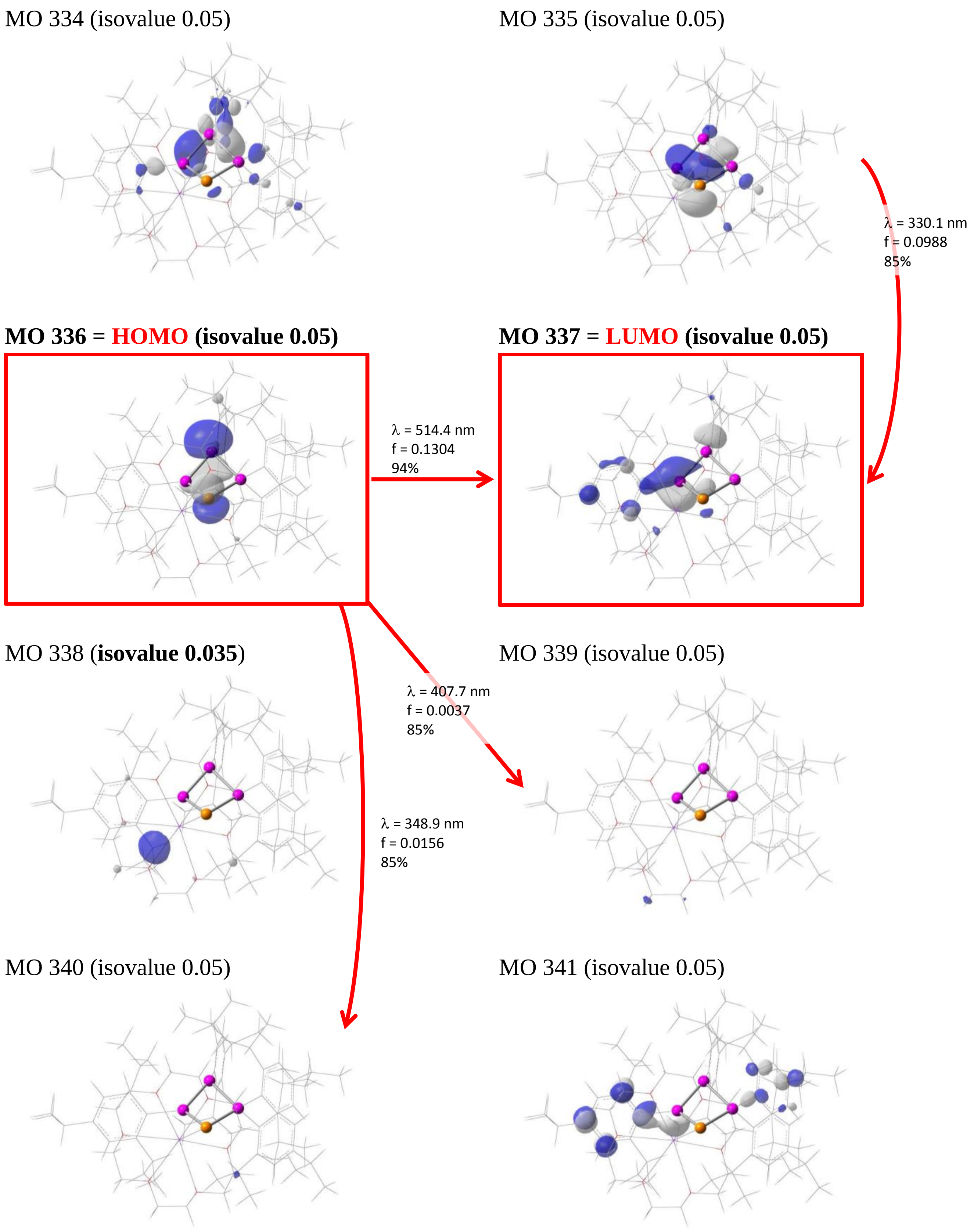

MO 334 (isovalue 0.05)
MO 335 (isovalue 0.05)
λ = 330.1 nm
f = 0.0988
85%
MO 336 = HOMO (isovalue 0.05)
MO 337 = LUMO (isovalue 0.05)
λ = 514.4 nm
f = 0.1304
94%
MO 338 (isovalue 0.035)
MO 339 (isovalue 0.05)
λ = 407.7 nm
f = 0.0037
85%
λ = 348.9 nm
f = 0.0156
85%
MO 340 (isovalue 0.05)
MO 341 (isovalue 0.05)

MO 342 (isovalue 0.05)

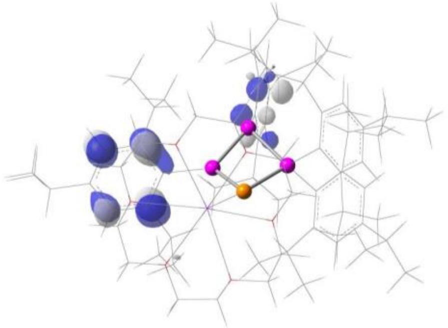

MO 343 (isovalue 0.05)

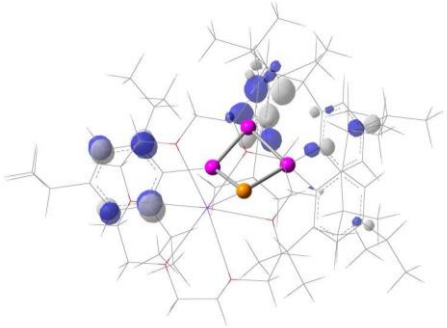

MO 344 (isovalue 0.05)

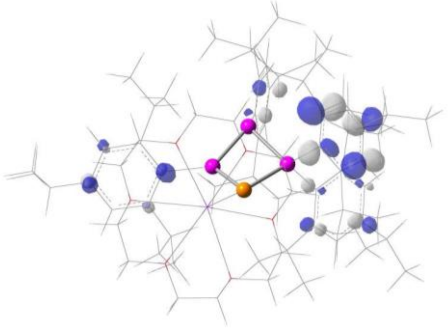

MO 345 (isovalue 0.05)

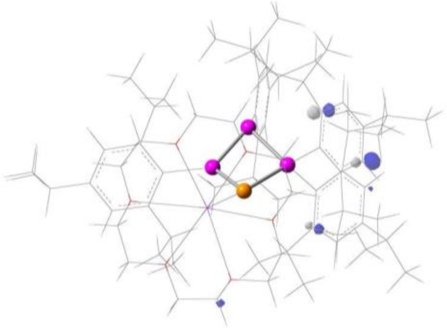

MO 346 (isovalue 0.05)

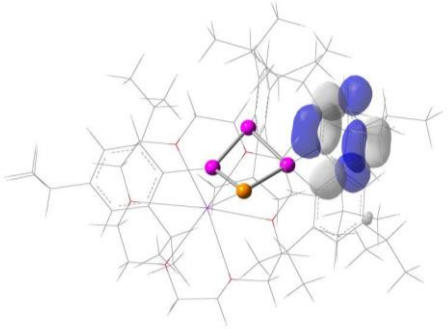

GIAO calculations for [K(18-crown-6)][**2**]$_{calc}$

Reference at: $SiMe_4$ (^{29}Si : 355.4917, ^{13}C: 190.2308);

PMe_3 (^{31}P: 391.7033 corrected to H_3PO_4 reference by subtracting 61 ppm)

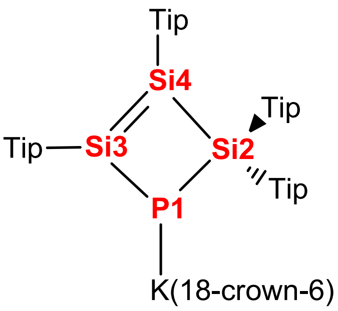

**Atom**	**δ_{exp} ([D_8]-THF)**	**δ_{calc} (scrf(cpcm,solvent=thf))**
P1	−55.7 ppm	−76.9 ppm (vs. H_3PO_4, 85%)
Si2	−14.9 ppm	−11.0 ppm
Si3	+193.1 ppm	+265.8 ppm
Si4	−39.9 ppm	−39.2 ppm

Excerpt from Gaussian09/GIAO output (corrected for atom order)

```
   1  P    Isotropic =   407.6499   Anisotropy =   270.3494
XX=   304.4439   YX=  -130.7151   ZX=   -98.0652
XY=  -109.6198   YY=   425.8525   ZY=   -15.4923
XZ=    33.7680   YZ=  -213.4940   ZZ=   492.6532
Eigenvalues:   202.1052   432.9616   587.8828
   2  Si   Isotropic =   366.5408   Anisotropy =   118.2020
XX=   357.0259   YX=    22.4052   ZX=   -49.1195
XY=    16.5732   YY=   309.9455   ZY=    47.2905
XZ=    -1.1276   YZ=    17.7166   ZZ=   432.6511
Eigenvalues:   291.8934   362.3869   445.3422
   3  Si   Isotropic =    89.6832   Anisotropy =   427.6442
XX=     5.0640   YX=   -80.3327   ZX=    24.2390
XY=  -120.5487   YY=  -100.9329   ZY=   -32.7934
XZ=   -34.9248   YZ=  -102.5812   ZZ=   364.9186
Eigenvalues:  -168.4647    62.7350   374.7793
   4  Si   Isotropic =   394.6634   Anisotropy =   135.4245
XX=   477.3045   YX=   -55.5189   ZX=   -21.2187
XY=     0.7770   YY=   360.6449   ZY=     3.1927
XZ=    54.7716   YZ=    13.4240   ZZ=   346.0407
Eigenvalues:   336.7990   362.2448   484.9464
```

NMR-Standards at ωB97XD/6-311g(d,p); scrf(cpcm,solvent=thf)

$\underline{SiMe_4}$ (NIF = 0)

```
Si                   0.00005   0.00003  -0.00001
C                    1.84855   0.33705   0.07691
H                    2.25063   0.5656   -0.91479
H                    2.06888   1.18755   0.72916
H                    2.39287  -0.5293    0.46485
C                   -0.63916  -0.38611   1.7258
H                   -0.46645   0.45152   2.40837
H                   -1.71443  -0.58858   1.71322
H                   -0.14015  -1.26506   2.14508
C                   -0.8863    1.5164   -0.67189
H                   -0.536     1.76608  -1.67802
H                   -1.96654   1.35099  -0.72739
H                   -0.71588   2.38919  -0.03429
C                   -0.32313  -1.46736  -1.1308
H                    0.18001  -2.36743  -0.76462
H                   -1.39288  -1.68791  -1.19674
H                    0.03943  -1.27292  -2.14475
```

```
     1  Si   Isotropic =   355.4917   Anisotropy =     0.1971
  XX=   355.5405   YX=     0.0273   ZX=    -0.0994
  XY=    -0.0436   YY=   355.4883   ZY=    -0.1858
  XZ=    -0.0329   YZ=    -0.0847   ZZ=   355.4463
  Eigenvalues:   355.3163   355.5357   355.6231
     2  C    Isotropic =   190.2308   Anisotropy =     8.0421
  XX=   195.3105   YX=     1.4401   ZX=     0.3062
  XY=     1.4479   YY=   187.7919   ZY=     0.0370
  XZ=     0.3567   YZ=     0.0614   ZZ=   187.5902
  Eigenvalues:   187.5215   187.5788   195.5923
     3  H    Isotropic =    31.9949   Anisotropy =     9.7793
  XX=    32.7245   YX=     1.5529   ZX=    -4.2199
  XY=     1.5047   YY=    28.5219   ZY=    -1.8541
  XZ=    -3.9671   YZ=    -1.8005   ZZ=    34.7383
  Eigenvalues:    27.9509    29.5193    38.5144
```

$\underline{PMe_3}$ (NIF = 0)

```
P                   -0.00004   0.00046  -0.60525
C                   -1.41978   0.79099   0.27979
H                   -2.35311   0.30592  -0.01573
H                   -1.31592   0.72353   1.3675
H                   -1.48838   1.84413  -0.00284
C                    1.39537   0.83324   0.27979
H                    1.29426   0.76254   1.36762
H                    2.3428    0.37681  -0.01646
H                    1.43192   1.88808  -0.00262
C                    0.02446  -1.62493   0.27931
H                   -0.85095  -2.21297  -0.00626
H                    0.91307  -2.18937  -0.01287
H                    0.02673  -1.50131   1.36713
```

```
   1  P    Isotropic =   391.7033   Anisotropy =    16.7347
XX=   402.6369   YX=     0.0424   ZX=     0.0594
XY=     0.0529   YY=   402.8496   ZY=    -0.2124
XZ=     0.0527   YZ=     0.1619   ZZ=   369.6235
Eigenvalues:   369.6234   402.6268   402.8598
   2  C    Isotropic =   171.9652   Anisotropy =    28.1018
XX=   178.9549   YX=   -10.7654   ZX=     6.8384
XY=   -10.6981   YY=   165.9889   ZY=    -3.6754
XZ=    11.7891   YZ=    -6.4517   ZZ=   170.9518
Eigenvalues:   159.9297   165.2662   190.6997
   3  H    Isotropic =    30.9512   Anisotropy =    10.4647
XX=    37.6026   YX=     1.2776   ZX=    -1.3876
XY=     1.2867   YY=    27.8974   ZY=    -0.8198
XZ=    -1.0305   YZ=    -0.6419   ZZ=    27.3537
Eigenvalues:    26.8380    28.0880    37.9277
```

8. References

[1] A. R. Jupp, J. M. Goicoechea, *Angew. Chem.* **2013**, 125, 10248–10251; *Angew. Chem. Int. Ed.* **2013**, *52*, 10064−10067.

[2] K. Leszczyńska, K. Abersfelder, A. Mix, B. Neumann, H.-G. Stammler, M. J. Cowley, P. Jutzi, D. Scheschkewitz, *Angew. Chem.* **2012**, *124*, 6891–6895; *Angew. Chem. Int. Ed.* **2012**, *51*, 6785–6788.

[3] *CrysAlisPro*, Agilent Technologies, Version 1.171.35.8.

[4] (a) G. M. Sheldrick in SHELXL97, *Programs for Crystal Structure Analysis (Release 97-2)*, Institut für Anorganische Chemie der Universität, Tammanstrasse 4, D-3400 Göttingen, Germany, 1998; (b) G. M. Sheldrick, *Acta Crystallogr. Sect. A* **1990**, 46, 467−473; (c) G. M. Sheldrick, *Acta Crystallogr. Sect. A* **2008**, *64*, 112−122.
